# Protocol for investigating intracellular microbial diversity using single-cell RNA-seq in immune cells of SARS-CoV-2-positive and recovered patients

**DOI:** 10.1016/j.xpro.2024.103546

**Published:** 2025-01-08

**Authors:** Jyoti Soni, Priyanka Mehta, Sunita Yadav, Partha Chattopadhyay, Rajesh Pandey

**Affiliations:** 1 Division of Immunology and Infectious Disease Biology, INtegrative GENomics of HOst-PathogEn (INGEN-HOPE) Laboratory, CSIR-Institute of Genomics and Integrative Biology (CSIR-IGIB), Mall Road, Delhi 110007, India; 2 Academy of Scientific and Innovative Research (AcSIR), Ghaziabad 201002, India

**Keywords:** classification description: bioinformatics, sequence analysis, genomics, sequencing, RNA-seq, microbiology

## Abstract

Intracellular microorganisms like viruses and bacteria impact immune cell function. However, detection of these microbes is challenging as the majority exist in a non-culturable state. This protocol presents detailed steps to investigate intracellular microbial diversity using single-cell RNA sequencing (scRNA-seq) in immune-cells of SARS-CoV-2-positive and recovered patients. We present a workflow from sample collection to library preparation, covering peripheral blood mononuclear cell (PBMC) isolation, single-cell labeling, cartridge priming, and cell lysis. We outline the steps for analyzing the scRNA-seq data, from data quality control (QC) to detection of intracellular microbes.

For complete details on the use and execution of this protocol, please refer to Yadav et al.^1^

## Highlights

• Protocol to isolate PBMCs and capture transcriptome from single cells
• Guidance on classifying unmapped sequencing reads generated from scRNA-seq
• Instructions for cell-specific quantification of microbial abundance
• Steps for differential microbial abundance analysis at the pseudo-bulk level

## Abstract (editor highlights)

Publisher’s note: Undertaking any experimental protocol requires adherence to local institutional guidelines for laboratory safety and ethics.

## Graphical abstract

**Figure:**
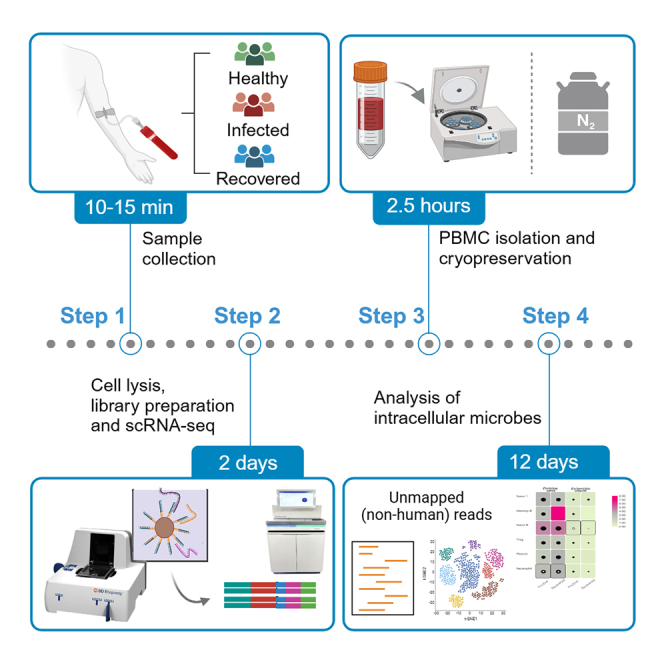

## Before you begin

The protocol below highlights detailed workflow of analyzing single-cell RNA sequencing (scRNA-seq) data to study traditionally non-culturable intracellular microbes at a resolution that was previously unattainable. The scRNA-seq approach allows for the simultaneous capture of host and microbial transcripts, providing a more extensive view of host-pathogen interactions within individual cells. This protocol innovatively applies scRNA-seq data, repurposing unmapped reads to reveal intracellular pathogens in specific immune cell populations in SARS-CoV-2 positive, recovered and healthy individuals. The applicability of this protocol can be expanded to other infectious diseases.

***Note:*** Care should be taken while isolating PBMCs from patient blood samples to perform single-cell lysis to preserve both host and microbial transcripts.

**CRITICAL:** Appropriate informed consent and permissions from the relevant institutions are required for the collection and use of human samples.

***Note:*** This tutorial uses BD Rhapsody single-cell RNA-seq dataset and uses intermediate files generated from running BD Rhapsody pipeline on Seven Bridges which requires users to create an account to run the analysis. We also mentioned alternative open-access tools that can be explored wherever possible.

### Institutional permissions

Conducting patient-related experiments requires strict adherence to local institutional guidelines for laboratory safety and ethical standards. Institutional ethical clearance for the study was obtained from both CSIR-IGIB, Delhi and Dr. D. Y. Patil Medical College, Hospital & Research Centre, Maharashtra. The studies involving human participants were reviewed and approved by CSIR-IGIB’s Human Ethics Committee Clearance (Ref No: CSIR-IGIB/IHEC/2020–21/01). The study was conducted following the guidelines of the Declaration of Helsinki. The patients/participants provided their written informed consent to participate in this study.

### Blood sample collection

**Timing: 10–15 min**

Collect blood samples from COVID-19 patients, healthy volunteers, and COVID-19 recovered individuals. In this protocol samples are collected at the Dr. D. Y. Patil Medical College, Hospital & Research Centre in Kolhapur, Maharashtra, India, using trained paramedical staff. Ensure all recovered samples are collected within a month of recovery and confirmed by negative RT-PCR (TRUPCR SARS-CoV-2 RT-qPCR Kit, catalog no 3B306, Ct value > 35).

Use BD Vacutainer CPT cell preparation tubes containing sodium heparin for sample collection. Isolate PBMCs from 5 mL of blood using the BD Vacutainer CPT cell preparation tube with sodium heparin.

### Preparation for running the code

#### R and RStudio installation

**Timing: 20–30 min**

1. The latest version of R can be found at https://cran.r-project.org/. This is the official website for the R programming language, where you can download the latest version and related packages.
2. The installation of RStudio is not mandatory to follow the protocol. However, it makes viewing and interacting with files, packages, objects in the environment, tables, and graphs. RStudio can be found at https://www.rstudio.com/. which can greatly enhance your R programming experience by providing a user-friendly interface for working with R scripts and data.
3. Installing packages used in this tutorial:

# List of CRAN packages

cran_packages <- c(

 
"tidyverse", "cowplot", "reshape2","dplyr","sctransform",

 
"pheatmap", "png", "RColorBrewer", "data.table","rtracklayer",

 
"Matrix","ggplot2","Seurat")

# Function to install CRAN packages if not already installed

installed_packages <- cran_packages %in% rownames(installed.packages())

if(any(!installed_packages)) {

 
install.packages(cran_packages[!installed_packages])

} else {

 
cat("All packages are already installed.\n")

}

# List of Bioconductor packages

BiocManager::install(c(

 
"edgeR", "S4Vectors","metagenomeSeq",

 
"SingleCellExperiment", "apeglm", "DESeq2","phyloseq"))

### Installation of tools/software before analysis

**Table:** 

Tool	Usage	Installation command
**Bcl2fastq**	Bcl2fastq is used for **demultiplexing** Illumina sequencing output files, converting the raw .bcl (base call) files into .fastq files. It separates reads from different samples according to their barcodes and generates the associated FASTQ files needed for downstream analysis. This tool also allows users to trim adapter sequences and filter out low-quality reads during the conversion process.	conda install -c bioconda::bcl2fastq
**FastQC**	FastQC is a tool used for performing **quality control checks** on raw sequencing reads in FASTQ format. It provides a visual report summarizing the quality metrics, including per-base sequence quality (Phred scores), GC content, presence of adapter sequences, and overrepresented sequences. It is commonly used as the first step in data analysis to assess the quality of sequencing data before proceeding to downstream analyses.	conda install -c bioconda::fastqc
**Trimmomatic**	Trimmomatic is a **read trimming tool** that removes low-quality bases from sequencing reads, cuts off adapters, and discards short reads. It helps improve the quality of the dataset by trimming or removing portions of reads that are likely to introduce errors in downstream analysis, ensuring that only high-quality reads are used.	conda install -c bioconda::trimmomatic
**STAR**	STAR (Spliced Transcripts Alignment to a Reference) is a **splice-aware aligner** used to map RNA-seq reads to a reference genome. It can accurately align reads across exon-exon junctions, which is essential for correctly mapping reads from spliced transcripts. It is typically used in RNA-seq workflows to quantify gene expression and annotate cell types based on mapped reads. Unmapped reads, which do not align to the human genome, can be further analyzed to explore for microbial or other non-human content.	conda install -c bioconda::STAR
**Kraken2**	Kraken2 is a **metagenomic classifier** that assigns taxonomic labels to DNA sequences by matching them to a reference database. It is often used to identify microbial content in a sample by classifying unmapped reads that do not align to the host genome. This tool can identify the presence of bacteria, viruses, fungi, and other microorganisms in sequencing data, making it useful for metagenomic and microbiome studies.	conda install -c bioconda::kraken2
**Sevenbridges-python**	Sevenbridges-python is a **Python SDK** for interacting with the Seven Bridges Genomics platform, particularly for workflows such as those on the BD Rhapsody platform, which is used for single-cell RNA sequencing. This tool allows users to automate the quantification of single-cell data, manage datasets, and run pipelines programmatically, integrating with the Seven Bridges cloud platform for scalable data analysis.	conda install -c bioconda sevenbridges-python
**AGAT**	Tool to convert gff files to gtf	conda install -c bioconda agat

***Note:*** Set up the “conda” package manager using the official link (https://conda.io/projects/conda/en/latest/user-guide/getting-started.html) for creating and activating the conda environment.

## Key resources table

**Table:** 

REAGENT or RESOURCE	SOURCE	IDENTIFIER
**Chemicals, peptides, and recombinant proteins**		

Ethyl alcohol, pure (200 proof, molecular biology grade)	Sigma-Aldrich	Cat#E7023-500ML
RPMI 1640	Thermo Fisher Scientific	Cat#11875093
Fetal bovine serum (FBS)	Thermo Fisher Scientific	Cat#26140079
L-glutamine	Thermo Fisher Scientific	Cat#A2916801
Penicillin-streptomycin	Thermo Fisher Scientific	Cat#15070063
Trypan blue solution, 0.4%	Thermo Fisher Scientific	Cat#15250061
1× PBS	Thermo Fisher Scientific	Cat#10010023
Fetal bovine serum, certified, heat inactivated, United States	Thermo Fisher Scientific	Cat#10082147
DMSO (dimethyl sulfoxide)	Sigma-Aldrich	Cat#276855
BD DRAQ7	BD Biosciences	Cat#BD 564904
Calcein AM	Thermo Fisher Scientific	Cat#C1430
BD stain buffer	BD Biosciences	Cat#554656
BD Rhapsody cartridge kit	BD Biosciences	Cat#633733
BD Pharmingen human BD Fc Block	BD Biosciences	Cat#564220
BD Vacutainer CPT	BD Biosciences	Cat# 362753
BD human single-cell multiplexing kit	BD Biosciences	Cat# 633781
BD Rhapsody WTA amplification kit	BD Biosciences	Cat# 633801
BD Rhapsody cDNA kit	BD Biosciences	Cat# 633773
AMPure XP	Beckman Coulter	Cat# A63881
Qubit dsDNA HS assay kit	Invitrogen	Cat#Q32854
Agilent high sensitivity DNA kit	Agilent	Cat#5067-4626
NovaSeq 6000 S2 reagent kit (200 cycles)	Illumina	Cat# 20040326

**Deposited data**		

Raw and analyzed data single-cell data	This study	GEO: GSE201088
Raw bulk RNA-seq data	This study	SRA: PRJNA816679; https://www.ncbi.nlm.nih.gov/bioproject/?term=PRJNA816679

**Software and algorithms**		

Kraken2 2.1.2	Wood et al.^2^	https://github.com/DerrickWood/kraken2
Pavian 1.2.0	Breitwieser et al.^3^	https://github.com/fbreitwieser/pavian
MetagenomeSeq 1.42.0	Paulson et al.^4^	https://github.com/HCBravoLab/metagenomeSeq
PhyloSeq 1.44.0	McMurdie et al.^5^	https://github.com/joey711/phyloseq
STAR 2.5.2b	Dobin et al.^6^	https://github.com/alexdobin/STAR/releases
Seurat 4.2.0	Hao et al.^7^	https://github.com/satijalab/seurat
BD Rhapsody WTA analysis pipeline	N/A	https://www.bdbiosciences.com/content/dam/bdb/marketing-documents/BD_Single_Cell_Multiomics_Analysis_Setup_User_Guide.pdf
ggpubr v.0.6.0	Kassambara et al.	https://github.com/kassambara/ggpubr
ggplot2 v.3.4.2	Steenwyk et al.^8^	https://github.com/tidyverse/ggplot2
tidyverse	Wickham et al.^9^	https://github.com/tidyverse
cowplot	Wilke	https://github.com/wilkelab/cowplot
reshape2	Wickham^10^	https://github.com/cran/reshape2
dplyr	Wickham et al.	https://github.com/tidyverse/dplyr
sctransform	Hafemeister and Satija^11^	https://github.com/satijalab/sctransform
pheatmap	https://CRAN.R-project.org/package=pheatmap (2012)	https://github.com/raivokolde/pheatmap
png	Urbanek (http://www.rforge.net/png/)	https://github.com/s-u/png
RColorBrewer	Neuwirth^12^	https://github.com/cran/RColorBrewer
data.table	Barrett et al.^13^	https://github.com/Rdatatable/data.table
rtracklayer	Lawrence et al.^14^	https://github.com/lawremi/rtracklayer
edgeR	Chen et al.^15^	https://github.com/OliverVoogd/edgeR
Matrix	Shabalin^16^	https://github.com/cran/Matrix
S4Vectors	Pagès (https://bioconductor.org/packages/S4Vectors)	https://github.com/Bioconductor/S4Vectors
SingleCellExperiment	Amezquita et al.^17^	https://github.com/drisso/SingleCellExperiment
apeglm	Zhu et al.^18^	https://github.com/azhu513/apeglm
DESeq2	Love et al.^19^	https://github.com/thelovelab/DESeq2

**Other**		

Themomixer (16°C–37°C, 1,200 rpm)	Eppendorf	Cat#5382000023
Vortexer	major supplier	N/A
Centrifuge	Eppendorf 5810R refrigerated centrifuge	Cat#5810R
Low retention filtered pipette tips (10, 200, 1,000 μL)	Axygen	N/A
Corning cell strainer 40 micron	Corning	Cat#431750
Water bath	Ambinova	N/A
Falcons 50 mL, 15 mL	Tarsons	Cat#546041 Cat#520060
DNA LoBind tubes, 1.5 mL	Eppendorf	Cat#0030.08051
CRYOCHILL 1° cooler	Tarsons	Cat#525000
CRYOCHILL vial star foot vials sterile	Tarsons	Cat#523193
Gilson PIPETMAN Tipack filtered tips, 100–1,200 μL	Thermo Fisher Scientific	Cat#F171803G
Gilson PIPETMAN Tipack filtered tips, 500–5,000 μL	Thermo Fisher Scientific	Cat#F161370G
BD Rhapsody express single-cell system	BD Biosciences	Cat#633707
BD Rhapsody scanner	BD Biosciences	Cat#633701
50 mL magnetic rack with adapter VP	V&P Scientific	Cat#VP 772FB-1 Cat#VP 772FB-1A
6-tube magnetic separation rack for 1.5 mL tubes	New England Biolabs	Cat#S1506S
Low-profile magnetic separation stand for 0.2 mL, 8-strip tubes	V&P Scientific	Cat#VP 772F4-1
Digital timer	Major supplier	N/A
Countess 3 automated cell counter	Thermo Fisher Scientific	N/A
Qubit 4.0 fluorometer	Thermo Fisher Scientific	Cat#Q33240
Bioanalyzer 2100 instrument	Agilent Technologies	Cat#G2939BA
NovaSeq 6000 system	Illumina	Cat# 20012850

## Materials and equipment

The key resources table lists all resources, materials, and software.

### Bioinformatics analysis

All bioinformatics analyses have been conducted on the CSIR-IGIB’s high-performance GPU-linux-based scientific computing server (CentOS 7.7) and a local workstation. A basic knowledge of scripting languages (bash, R, and Python) is required to understand and apply this protocol. Software and scripts used in this protocol are mentioned (See the software and algorithms section of the key resources table).

### Computational requirements for analysis

Basic installation requirements:

• Memory = 400–600 GB.
• Cores = 4–8.
• Compute nodes = 40–80.
• Operating system (Windows, Linux, Mac).
• GPUs (V100)-Linux-based (CentOS 7.7) compute cluster.

***Note:*** A computer with a Linux and network connection is required. The RAM requirement depends on the number of samples to be analyzed. 16 GB RAM should be sufficient for an initial analysis. Based on the availability of RAM, analysis time may fluctuate (fast).

***Note:*** All the codes used in this study can also be available in github: https://github.com/INGEN-HOPE/Single-cell-microbial-Analysis.

## Step-by-step method details

### PBMC isolation

**Timing: 2.5 h**

This major step describes the isolation of peripheral blood mononuclear cells from the blood samples of healthy, hospital-admitted SARS-CoV-2 positive, and recovered patients.

***Note:*** Thaw 1× PBS (filtered) and FBS at 25°C before starting the experiment. To prepare 50 mL of freezing media, mix 45 mL of FBS with 5 mL of DMSO. (See Tables 1 and 2.)

1. Blood collection and PBMC isolation.
  Collect blood into the tube using standard venipuncture techniques. Ensure the BD Vacutainer CPT Tube is at 25°C and properly labeled for patient identification.
  ***Note:*** In this experiment, 3–5 mL of blood was collected from each patient in the BD Vacutainer CPT Tubes. For standard BD Vacutainer CPT Tubes, typically, up to 8 mL of blood can be collected.
  a. Store the tube upright at 25°C until centrifugation.
    ***Note:*** Centrifuge within 2 h to ensure proper barrier formation.
  b. Just before centrifugation, gently invert the tube 8–10 times to remix the blood.
  c. Place the tube in a horizontal rotor (swing-out head) centrifuge.
  d. Centrifuge at 25°C for at least 15 min at 1500–1800 × *g.*
    ***Note:*** Do not exceed 2000 × *g* to avoid tube breakage.
  e. After centrifugation, locate the whitish mononuclear cell layer just below the plasma layer.
  f. Aspirate approximately half of the plasma without disturbing the cell layer.
  g. Use a sterile pipette to collect the mononuclear cell layer and transfer it to a 15 mL conical centrifuge tube.
    ***Note:*** In general, from 5 mL of blood, 3.5–4 mL of plasma layer is expected, while around 0.3–1 mL of mononuclear cell layer is expected. This can vary depending on the cell yield, which is influenced by factors such as donor age, gender, blood sample quality, and other clinical parameters.
  h. Add 1× PBS to the cell suspension to bring the volume to 15 mL.
  i. Cap the tube, and mix by inverting 5 times.
  j. Centrifuge at 300 × *g* for 15 min and aspirate the supernatant carefully.
  k. Resuspend the pellet, add 1× PBS to bring the volume to 10 mL.
  l. Cap the tube, and mix by inverting 5 times.
  m. Centrifuge at 300 × *g* for 10 min and aspirate the supernatant carefully.
  n. Resuspend the cell pellet in the cryopreservation medium for cryopreservation.
  o. Dissolve the final PBMC pellet in 1 mL of freezing media (90% FBS and 10% DMSO) gently.
  p. Transfer to a labeled cryotube.
    ***Note:*** The yield of PBMCs can vary based on the individual donor's health, age, and other factors. On average, one can expect to obtain approximately 1–2 million PBMCs per mL of blood.^20^
  q. Store in a cryocooler at −80°C.
  r. Transfer these cryotubes to liquid nitrogen (−196°C) within one week until further use.
    ***Note:*** Do not do excess centrifugation, as it can lead to tube breakage and uneven separation of whole blood.

**Table 1:** Reagents required for freezing media preparation

Reagent	For 1 rxn.	Storage
FBS	45 mL	−20°C
DMSO	5 mL	25°C
Total	50 mL	–

**Table 2:** Reagents required for PBMC isolation

Reagent	For 1 rxn.	Storage
1× PBS	25 mL	25°C
Freezing media (FBS+DMSO)	1 mL	4°C

### Cell defrosting

**Timing: 30 min**

This step is required for defrosting and reviving the cryopreserved cells to maintain viability.

2. Media preparation for cell revival.
  a. Keep the following reagents at 37°C.
    i. RPMI 1640 (11875093) - 88 mL.
    ii. Fetal bovine serum (FBS) - 10 mL.
    iii. L-Glutamine - 1 mL.
    iv. Penicillin-Streptomycin - 1 mL.
  b. Add 10% FBS, 1% L-Glutamine, and 1% penicillin-streptomycin to RPMI 1640 to make complete media.
    ***Note:*** Pre-heat the water bath at 37°C.
  c. Bring out an empty cryocooler from −80°C.
  d. Immediately transfer the stored PBMC samples from liquid nitrogen to the cryocooler.
    ***Note:*** Thaw samples at 25°C inside the cryocooler only.
  e. Once the samples are thawed properly (no ice pellet should be left), place the PBMC vials into a 37°C water bath for 1–2 min and proceed further.
  f. For each vial, remove the PBMCs from the water bath until a small ice pellet remains.
  g. Slowly add 1 mL of warm complete media to the PBMCs in the cryovial in a dropwise manner.
  h. Transfer the contents of the vial dropwise to a 15 mL conical tube with 10 mL of pre-warmed complete media.
  i. Using 1 mL of warm complete media, rinse the vial to ensure collection of all cells and add to the 15 mL conical tube.
  j. Centrifuge cells at 400 × *g* for 10 min.
  k. Decant the supernatant and wash twice with 5 mL of PBS.
  l. Resuspend the pellet for each sample in 1 mL of warm PBS.
  m. Count cells using trypan blue to ascertain cell viability (see below).
  n. Filter cells through a 40 μm cell strainer into a 50 mL Falcon tube with a strainer cap.
  o. Take an aliquot containing the desired number of cells for analysis from each sample and transfer them to new 1.5 mL LoBind tubes.
    ***Note:*** It is recommended to start with at least three times the cell number that should be later loaded on the cartridge. From 1 mL of blood, one can expect 1 million to 2 million cells.^20^
  p. Centrifuge the tubes at 400 × *g* for 5 min and remove the supernatant.
  q. Resuspend the cell pellets in 500 μL of BD stain buffer.
    ***Note:*** The process of filtering samples with low volume is not mandatory; however, BD Biosciences recommends filtering the final sample before it is inserted into the cartridge. Additionally, if RBC contamination is visible, an RBC lysis step is recommended after first PBS washing.

### Viability check

**Timing: 5–10 min**

Here, we describe steps for cell count and viability check.

Cell counting is an important step as it determines the concentration and viability of cells in a sample, crucial for deciding if the sample is suitable for further use.

3. Cell Counting.
  a. For checking, make an aliquot of 10 μL of the sample.
  b. Add 10 μL of trypan blue and mix before loading into the cell counter chamber slide.
  c. Load 10 μL of sample and dye mix into the slide groove.
  d. Place the slide inside the Countess Automated Cell Counter.
  e. Press the “Count cell”.
  f. Note the cell count and percentage of viable cells for further consideration.

***Note:*** While performing viability checks on the cells, ensure the rest of the sample remains at 37°C in the water bath.

### Sample multiplexing

**Timing: 30–45 min**

Here, we describe steps for labeling each sample with sample tags.

4. Sample Preparation for Multiplexing.
  a. Proceed with the sample if the viable cell count is greater than 75%.
  b. Transfer the cell suspension into a fresh 1.5 mL Lobind tube.
  c. Centrifuge at 400 × *g*, 5 min at 25°C.
  d. Discard the supernatant and add 200 μL of stain buffer.

This protocol describes the tagging of each sample with unique nucleotide sequences containing antibodies for multiplexing.

5. Sample Tagging.
  a. Make a spreadsheet and mark which sample is to be labeled with which sample tag.
  b. Take out the sample tag vials according to the number of samples being processed.
    ***Note:*** Each vial contains 20 μL of sample tag. From the cell suspension prepared in Step 4 (d), pipet 180 μL of the suspension into each vial containing 20 μL of sample tag.
  c. Gently mix the contents to ensure the sample tag is evenly distributed throughout the cell suspension.
  d. Incubate for 20 min, then transfer the mixture to a 1.5 mL tube for more efficient handling of the cell suspension.
  e. Add 1 mL of stain buffer, and centrifuge at 400 × *g* for 5 min at 25°C.
  f. Now, discard the supernatant, without disturbing the pellet.
  g. Add 500 μL of stain buffer again, and centrifuge at 400 × *g* for 5 min at 25°C.
  h. Discard the supernatant and add 500 μL of stain buffer or 1 mL based on the size of the pellet.
    ***Note:*** For low abundance samples, resuspend samples in 200–300 μL of BD stain buffer.
  i. Count the cell number of each sample, as described above.
  j. Pool the cells of all samples in equal numbers and adjust the volume with BD Sample buffer to make the final volume 610 μL.
    ***Note:*** While counting the cells, ensure the remaining samples are kept on ice until further processing. Accurate cell counting is critical for obtaining an equal distribution of cells among the different samples in the final cell mixture. If the volume after pooling exceeds 610 μL, consider centrifuging one more time, removing the supernatant, and resuspending in 610 μL of sample buffer.
  k. Pool all the sample-tagged cell suspension in a single 1.5 mL Lobind tube, ensuring an equal number of cells from each sample.
  l. After pooling, centrifuge again at 400 × *g*, for 5 min at 25°C.
  m. Prepare the Fc block master mix by pipetting the following reagents into a new 1.5 mL LoBind tube on ice. (See Table 3.)
    ***Note:*** (Optional) For samples that contain myeloid and B lymphocytes, it is recommended to block non-specific Fc receptor-mediated false positive signals.

**Table 3:** Fc block master mix

Component	1 sample (μL)	1 sample + 10% overage (μL)
BD Stain Buffer	95.0	114.0
Human Fc block	5.0	6.5
Total	100.0	110.0
  n. After centrifugation at step 5 (L), discard the supernatant.
  o. Add 100 μL of cold staining buffer.
  p. Pipette mix and add 100 μL of Fc block master mix to the cell suspension.
  q. Incubate cells on ice for 10 min. Meanwhile, spin Ab-Oligos for further use.
  r. Prepare AbSeq antibody labeling master mix on ice. (See Table 4.)

**Table 4:** AbSeq labeling master mix

Component	1 sample (μL)	1 sample + 10% overage (μL)
Per BD AbSeq ab-oligo (N = No. of Ab-oligos)	2.0	2.2
Stain buffer	100-(2.0∗N)	110-(2.2∗N)
Total	100.0	110.0
  s. Pipette the following reagents into a 1.5 mL lobind tube.
    ***Note:*** BD AbSeq oligo-coupled antibodies are optimized for use at a final concentration of 1:100. Thus, 2 μL of each antibody are mixed, and depending on the number of antibodies used, stain buffer is added to reach a volume of 100 μL of the antibody mix.
  t. After incubation, add 100 μL of BD AbSeq labeling master mix.
  u. Incubate for 60 min on ice.
  v. Add 1 mL of stain buffer to the labeled cells and pipet mix for the washing step.
  w. Centrifuge at 400 for 5 min, 4°C.
  x. Discard the supernatant and repeat this step two to three times.
  y. Resuspend the pellet in 610 μL of cold staining buffer.
  z. Count the cells as mentioned in the above procedure.
  aa. Place the cells on ice and proceed for single-cell capture.
    ***Note:*** To pool cells for single cell sorting and lysis with the BD Rhapsody cartridge, which can accommodate approximately 20,000–40,000 cells. For instance, if you have 6 samples, you will combine 5,000 cells from each sample. After pooling, adjust the final volume to 610 μL by adding the BD sample buffer as required.

### Preparing the cartridge

**Timing: ∼2 h**

This section describes cell loading and capturing of single cells onto the BD Rhapsody Cartridge (RC) and subsequently cDNA synthesis and exonuclease treatment.

***Note:*** For priming and treating the BD Rhapsody Cartridge and further library preparation, we followed an already published star protocol provided by Erickson et al., for which the link is given below.

6. Treating the surface of the cartridge.
  a. Allow these reagents to equilibrate at 25°C for ≥30 min before use:
    i. Cartridge Wash Buffer 1 (Cat# 650000060).
    ii. Cartridge Wash Buffer 2 (Cat#650000061).
  b. Place these reagents on ice:
    i. Sample Buffer (Cat# 650000062).
    ii. 1 M DTT (Cat#650000063).
    iii. Lysis Buffer (Cat#650000064).
    iv. DRAQ7 (protected from light).
    v. Calcein AM.
  c. Review pipette settings (P1200M and P5000M) and operation.
  d. Prime and treat BD Rhapsody cartridge.
  e. The sample loading station “Front” Slider position should be “WASTE” and the side slider position should be “0”.
  f. Set the pipette in Prime/Treat mode and follow the table given below. (See Table 5.)
    ***Note:*** Make sure samples remain in the cold sample buffer.

**Table 5:** Prime treat requirements

Step no.	Material to load	Volume(μL)	P1200M	Incubation period
1.	100% Ethyl alcohol	700	Prime/Treat	NA
2.	Air	700	Prime/Treat	NA
3.	Cartridge wash buffer 1 (RT)	700	Prime/Treat	1 min
4.	Air	700	Prime/Treat	NA
5.	Cartridge wash buffer 1 (RT)	700	Prime/Treat	10 min
6.	Air	700	Prime/Treat	NA
7.	Cartridge wash buffer 2 (RT)	700	Prime/Treat	< 4 h
  g. Add 3.1 μL of 2 mM Calcein AM and 3.1 μL of 0.3 mM DRAQ7 to 620 μL cell suspension (1:200) in the cold sample buffer.
  h. Gently pipette-mix and incubate at 37°C in the dark for 5 min.
  i. Filter cells through a 50 mL Falcon tube with a cell strainer cap.
  j. Gently pipette 10 μL in INCYTO disposable hemocytometer and count cells immediately using the scanner.
    ***Note:*** Keep remaining cells on ice and protect them from light.
  k. Insert the hemocytometer into the hemocytometer adapter and tap “Scan”.
  l. Place the adapter on the scanner tray and “continue”.
  m. Select the protocol and select or enter the experiment name, sample and username.
  n. Tap slide A or slide B (depending on where the cells are loaded) and start slide A or slide B scan.
  o. Once the scan completes, click OK.
  p. Tap “Eject” to remove the adapter, then tap “done”.
  q. Tap on analysis and then experiment name to view total cell concentration and cell viability.
    ***Note:*** If the cell concentration is < 1000 cells/μLl proceed to the next step. Otherwise (>1000 cells/μL) dilute in a Cold sample buffer to ∼200–800 cells/μL. Count again and proceed to the next step.
  r. Use the sample calculator and get stock cell and buffer volume to prepare the final pool of 650 μL.
7. Loading cell suspension.
  a. Load the cartridge with the material listed in Table 6 using the P1200M pipette.
    ***Note:*** Pipette-mix cell suspension with a manual 1000 μL pipette before aspiring through an automated pipette.

**Table 6:** Cell loading requirements

Material to load	Volume (μL)	Pipette mode
Air	700	Prime/Treat
Cell suspension	575	Cell load^a^

a Press the pipette button once to aspirate 40 μL air, and then immerse the tip in cell suspension, press the button again to aspirate 575 μL of cold cell suspension.
  b. Dispense 615 μL of air and cold cell suspension.
  c. Incubate at 25°C for 15 min before tapping.
  d. Start cell load scan.
  e. Image the cells in the cartridge and perform the scanner step: “Cell load”.
  f. After scan completion, tap “OK” and “Eject”. Remove the cartridge and tap “done”.
    ***Note:*** Keep cell capture beads on ice before use and prepare cell capture beads during 15 min of incubation after cell loading.
8. Preparing cell capture beads for loading.
  a. Place cell capturing beads on the magnet for 1 min and remove the storage buffer.
  b. Then remove the tube from the magnet and resuspend in 750 μL of cold sample buffer into the tube, pipette mix and place.
  c. Set the P1200M pipette on prime treat mode.
  d. Put back the cartridge on BD Rhapsody Express.
9. Bead loading.
  ***Note:*** Mix beads immediately before loading to ensure proper suspension. Also, recheck the pipette mode. (See Table 7.)
  a. Incubate the cartridge at 25°C for 3 min.
  b. Perform scanner step “bead load”.
  c. After scan completion, place the cartridge back on BD Rhapsody Express.

**Table 7:** Bead loading requirements

Material to load	Volume (μL)	Pipette mode
Air	700	Prime/Treat
Cell capture beads	630	Bead Load
10. Bead Washing.
  a. Load the cartridge with the materials listed in Table 8 using a P1200M pipette.

**Table 8:** Bead washing requirements

Material to load	Volume (μL)	Pipette mode
Air	700	Wash^a^
Cold sample buffer	700	Wash
Air	700	Wash
Cold sample buffer	700	Wash

a Press the pipette button once to aspirate 720 μL of volume and insert the tip into the cartridge to dispense 700 μL or air or liquid. Remove the pipette and dispense the remaining air.
  b. Prepare the cell lysis buffer by adding 75 μL of 1 M DTT to one 15 mL lysis buffer bottle.
  c. Briefly vortex lysis mix and place it on ice.
  d. Place the cartridge on the express instrument.
  e. Now move the “left slider” to “lysis” and the front slider position to “waste”.
  f. Load the following materials into the cartridge. (See Table 9.)

**Table 9:** Cell lysis requirements

Material to load	Volume (μL)	Pipette mode
Lysis buffer with DTT	550	Lysis
  g. Incubate at 25°C for 2 min.
  h. Place a 5 mL lo-bind tube in the BD Rhapsody Express instrument drawer.
    ***Note:*** Use the lysis buffer prepared with DTT within 24 h and do not exceed the incubation time for optimal performance. Also, ensure that the P5000M pipette is set to “Retrieval mode”.
  i. Move the front slider to “beads” on “Express instrument” and the left slider to “Retrieval.”
  j. Leave the retrieval magnet in a down position for 30 s, and aspirate 5000 μL of lysis buffer with DTT using P5000M.
  k. Press down on the P5000M pipette to seal against the gasket.
  l. As 30 s get over move the left slider to the middle position ‘o’ and immediately load 4950 μL lysis buffer with DTT.
  m. Remove the pipette from the gasket and purge the tip.
  n. Move the front slider to “open”.
  o. Place the 5 mL Lo-bind tube on a 50 mL magnetic separation rack with a 15 mL clear acrylic cylinder adapter for 1 min (perform scanner step during this 1 min).
  p. Now, discard 4 mL of lysis buffer leaving 1 mL of supernatant without disturbing the beads.
  q. Remove the tube from the magnet. Gently pipette mix beads and transfer to a new 1.5 mL lo-bind tube.
    ***Note:*** If beads are left in the 5 mL tube, consider adding 500 μL of lysis buffer with DTT. Rinse the tube and transfer the contents to a 1.5 mL Lo-Bind tube.
  r. Place the tube on a 1.5 mL magnetic stand for ∼2 min and remove the supernatant.
  s. Remove the tube from the magnet and pipet 1.0 mL cold bead wash buffer.
  t. Pipette mix and place on ice for 2 min.
  u. Remove the supernatant, again add 1 mL cold bead wash buffer into the tube and place on ice.
    For reverse transcription, treatment with Exonuclease I on the cell capture beads and complete library preparation, and sequencing we followed an already published star protocol. It can be accessed by clicking on: (Domenico et al.).^21^

### Data analysis and interpretation

**Timing: ∼4–8 h**

**Timing: ∼2 days (for step 13)**

To download the data associated with the paper, “Single Cell Multiomics of Healthy, COVID-19 positive and Recovered Individuals”, use the GEO ID given in the paper, GSE201088.

11. Downloading the dataset or directly using sequencing dataset.
  a. Navigate the NCBI GEO FTP site (https://ftp.ncbi.nlm.nih.gov/geo/) to the series/ folder.
  b. Find the GSE20nnn/ directory.
  c. Enter the GSE201088/ folder.
  d. The data files available are in the suppl/ directory. If we choose to download all associated data, we can download the entire suppl/ directory.
  e. Use the wget command followed by the link to the suppl/ directory (right-clicking and choosing ‘Copy Link Address’).

**Figure:**
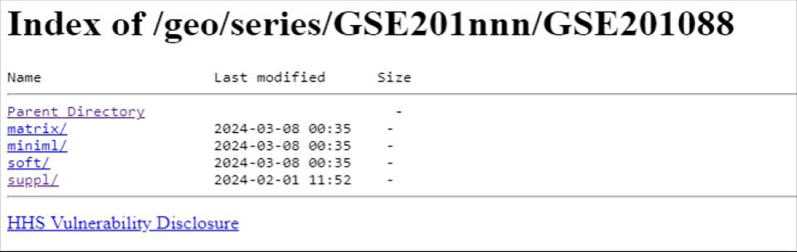

    #create a directory
    mkdir scRNAseq
    cd scRNAseq
    #download supplementary files
    wget --recursive --no-parent -nd https://ftp.ncbi.nlm.nih.gov/geo/series/GSE201nnn/GSE201088/suppl/GSE201088_RAW.tar
  f. After the download is complete, untar the file using the following commands. You can directly download associated metadata files. This will contain Expression_Data and RSEC_MolsPerCell files for each batch which will be used for cell annotation later.
    tar -xvf GSE201088_RAW.tar
    for all in ∗.gz; do gunzip $all; done
    ***Note:*** To download the raw sequence fastq files SRA toolkit will be required.
  g. Install SRA toolkit binaries for your specific OS available https://github.com/ncbi/sra-tools/wiki/02.-Installing-SRA-Toolkit. To download the fastq files, run the following command for selected SRA Run ID from SRA Run selector. Repeat for each SRA Run ID separately or follow the tutorial available https://erilu.github.io/python-fastq-downloader/ for batch download.
    prefetch SRR18827616
    fastq-dump SRR18827616.sra
    ***Note:*** If prefetch throws an error, increase max memory using --max-size command. Each fastq file size may range from 90–120 GB. This step will take some time depending on download speed. So, feel free to grab a coffee.
    ***Note:*** The intermediate files generated in this tutorial may be memory intensive.
    The R1 of both BD and 10× data contains the cell label and UMI indexes which will be used to identify cell counts whereas the R2 contains the cDNA which will be used for mapping to the human reference genome.
    ***Note:*** Reads QC/Trimming
    **Run** .**FastQC** to obtain information on per-base quality and adapter contamination.
    Trimming Procedures:
     • **Read1**: Trim bases beyond the cell label and UMI identification, taking into account the bead version used.
     • **Read2**: Trim bases with poor quality (Phred score < 20) and remove any template switch oligos (TSOs) present, such as TATGCGTAGTAGGTA or GTGGAGTCGTGATTATA.
12. Alignment to the human genome.
  a. The star index for the latest human genome or prebuilt human genomes available for both BD and 10× data can be used for performing alignment to the human genome. Alignment will be performed using STARsolo.
  b. First, we will build the human reference genome: For that we need the Primary assembly annotation gtf and fasta sequences from Ensembl or Gencode.
    #Download human gtf and fasta
    wget https://ftp.ebi.ac.uk/pub/databases/gencode/Gencode_human/release_46/gencode.v46.primary_assembly.annotation.gtf.gz
    wget https://ftp.ebi.ac.uk/pub/databases/gencode/Gencode_human/release_46/GRCh38.primary_assembly.genome.fa.gz
    #Build STAR index
    STAR --runThreadN 20 --runMode genomeGenerate --genomeDir STAR --genomeFastaFiles GRCh38.primary_assembly.genome.fa --sjdbGTFfile gencode.v46.primary_assembly.annotation.gtf --genomeSAsparseD 3
    (See Table 10.)

**Table 10:** STAR parameters and description

Parameter	Description
--runMode genomeGenerate	Used to build genome index from fasta and gtf files
--genomeDir STAR	Output directory
--sjdbGTFfile gencode.v46.primary_assembly.annotation.gtf	Path to gtf file
--genomeFastaFiles GRCh38.primary_assembly.genome.fa	Path to fasta file
-genomeSAsparseD	Sets the sparsity parameter for the suffix array. **NOTE:** Addition of `--genomeSAsparseD 3′ option, generates a sparse suffix array, optimizing memory usage while also reducing speed by ∼30%–40%. Alternatively, cellranger mkgtf can also be used to create a reference for 10× data. Cellranger reference annotations provide filtered biotypes which can also be used for indexing.
    Output:

**Figure:**
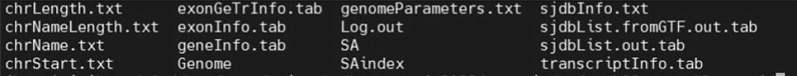
  c. Criteria for a valid R1 and R2 pairs:
    i. **Read2:** The mapping strategy includes aligning to both introns and exons to enhance mapping sensitivity and increase molecular counts or the number of genes detected per cell. A valid alignment requires that the sum of CIGAR alignment matches be at least 25, with reads uniquely aligned to either an exon or an intron in the reference, and not aligning to phiX174, which serves as a control in the library.
    ii. **Read1:** Valid reads must include identified cell label sequences (CLS) and a UMI sequence that contains no 'N' bases.
      #Aligning BD Rhapsody WTA data
      /path/to/STAR --runThreadN 40 \
      --genomeDir /path/to/STAR/ \
      --readFilesIn <Sample>_R2.fastq.gz <Sample>_R1.fastq.gz \
      --readFilesCommand zcat \
      --soloType CB_UMI_Complex \
      --soloCBmatchWLtype 1MM \
      --soloCBposition 0_0_0_8 0_13_0_21 0_26_0_34 \
      --soloUMIposition 0_35_0_43 \
      --soloCBwhitelist CL1S.txt CL2S.txt CL3S.txt \
      --outFileNamePrefix ./star_solo \
      --outSAMattributes CB UB \
      --outSAMtype BAM SortedByCoordinate
      (See Table 11.)
      ***Note:*** --readFilesIn first R2 should be mentioned then R1. R2 contains the captured sequence and R1 contains the CB and UMI. For aligning 10× reads the codes are modified as following:
      #Aligning 10× data
      STAR --runThreadN 40 \
       --genomeDir <path/to/STARindex> \
       --readFilesIn read1.fq read2.fq \
       --soloType CB_UMI_Simple \
       --soloCBstart 1 \
       --soloCBlen 16 \
       --soloUMIstart 17 \
       --soloUMIlen 10 \
       --outFileNamePrefix <sample_name>/ \
       --outSAMattributes CR UR CY UY CB UB \
       --outSAMtype BAM SortedByCoordinate
      ***Note:*** The read files are input in the Read1 Read2 order in this case. Alternatively, cellranger can be used for 10×.

**Table 11:** STARsolo parameters and description

Parameter	Description
--runThreadN 40	Number of threads used for alignment, in this case 40 threads are utilized for faster performance
--genomeDir /path/to/STAR/	Directory where the STAR genome index is stored. NOTE: The genome index is the same as for normal STAR runs.
--readFilesIn <Sample>_R2.fastq.gz <Sample>_R1.fastq.gz	Specifies the input paired-end read files, where R1 is forward and R2 is reverse. **Note:** first R2 should be mentioned then R1 in this case.
--soloType CB_UMI_Complex	Complex barcodes are activated using CB_UMI_Complex Simple barcodes are activated using CB_UMI_Simple
--soloCBposition 0_0_0_8 0_13_0_21 0_26_0_34	Defines positions for cell barcodes on the read, this only works with --soloType CB_UMI_Complex, Format: startAnchor_startDistance_endAnchor_endDistance **0_0_0_8**: The first barcode starts at the very beginning of the read (position 0) and spans the first 8 bases (ends at position 8). **0_13_0_21**: The second barcode starts at position 13 of the read and spans up to position 21 (total of 9 bases). **0_26_0_34**: The third barcode starts at position 26 and ends at position 34 of the read (also 9 bases long).
--soloUMIposition 0_35_0_43	Defines the positions of the UMI (Unique Molecular Identifier) on the read sequence.
--soloCBwhitelist CL1S.txt CL2S.txt CL3S.txt	Specifies the whitelists for known cell barcodes, using multiple whitelist files for different channels or libraries. NOTE: For cell barcodes use https://teichlab.github.io/scg_lib_structs/methods_html/BD_Rhapsody.html to find CLS1-3 and save it as CLS1.txt, CLS2.txt and CLS3.txt
--soloCBmatchWLtype 1MM	Allows up to 1 mismatch between the cell barcode and the whitelist of known barcodes.
--outSAMattributes CB UB	Bam tags: Includes Corrected cell barcode (CB) and UMI barcode (UB) attributes in the output SAM/BAM files.
--outSAMtype BAM SortedByCoordinate	Specifies the output file type (BAM) and sorting method (Sorted by genomic coordinates). NOTE: SortedByCoordinate must be used with --outSAMattributes
--soloCellFilter EmptyDrops_CR	Uses the CellRanger EmptyDrops method to filter out empty droplets.
--soloMultiMappers EM	STARsolo offers several algorithms for handling multi-mapped UMIs: EM (Expectation-Maximization): Uses maximum likelihood estimation to assign multi-mapped reads to genes, optimizing accuracy based on the gene expression model from unique and multi-mapped reads.

For additional parameters please refer to the STARsolo manual.
  d. For cell-filtering, additional curated options for multi-gene mapping can be included based on the sequencing chemistry used from the link.
    ***Note:*** To correct for errors in cell barcodes a user-input whitelist can additionally be added using --soloCBwhitelist <whitelist> command. To generate the whitelist UMI-tools is an option.
    ***Optional:*** Generating Whitelist.
    While mapping error correction and demultiplexing of cell barcodes using user-input whitelist.
    For alignment we need to first generate a whitelist of cell barcodes to identify cells using UMI-tools. (See Table 12.)
    #generating whitelist
    umi_tools whitelist --stdin CSP_R1_fastq.gz --extract-method=regex --bc-pattern=<barcode_pattern> --set-cell-number=<n> --log2stderr > whitelist.txt
    <n> will be the estimated number of cells.

**Table 12:** Regex pattern for extracting whitelist of UMI from BD and 10× data

<barcode_pattern>	Sequencing chemistry
(?P < cell_1>.{9})(?P < discard_1>GTGA)(?P < cell_2>.{9})(?P < discard_1>GACA)(?P < cell_3>.{9})(?P < umi_1>.{6,9})T{3}.∗	BD Rhapsody
(?P < cell_1>.{16})(?P < umi_1>.{10})	10× Chromium

The complete explanation of the regex pattern used above is available at https://umi-tools.readthedocs.io/en/latest/regex.html#regex-regular-expression-mode.
  e. Next, we will extract unmapped reads to check for metagenomic reads presence.
    #Extracting unmapped reads
    samtools view -u -f 4 <sample_name>_starsoloAligned.sortedByCoord.out.bam > unmapped.bam
    #Convert bam to fastq for classification
    samtools bam2fq unmapped.bam | gzip > <sample_name>.fastq.gz
13. Taxonomic classification using kraken2.
  Taking the reads unmapped to the human genome, we will run Kraken2 to check for presence of the microbial diversity at the bulk level. To select specific species with highest abundance throughout the sample set for downstream single-cell level analysis.
  For the kraken2 tool installed previously, download the maxikraken2_1903_140 GB database following the tutorial available https://lomanlab.github.io/mockcommunity/mc_databases.html.
  ***Note:*** If there is limitation of storage, kraken2-microbial (September 2018, 30 GB) can also be used.
  Download the database from the Kraken2 website, which contains bacteria, archaea, and viral reference sequences. Kraken2 map reads with taxa using k-mers from the genomic database and assigns taxonomy.
  a. Classification. (See Table 13.)
    kraken2 --threads 20 --db index --report < sample_name>.report --output <sample_name>.output --gzip-compressed <sample_name>.fastq.gz
    i. Change the <sample_name> with input fastq file names.
    ii. Run this command for each sample to generate the report file for all the samples and visualize using the Pavian tool (in R studio) to see the classified and unclassified microbial reads.
    iii. R1 was not used in this step as it contains cellular barcodes.

**Table 13:** Kraken parameters and description

Parameter	Description
--db index	Specifies the path to the Kraken2 database to use for classification. Replace index with your actual database path.
--report <sample_name>.report	Defines the file to which Kraken2 will write the classification report, summarizing the results.
--output <sample_name>.output	Specifies the file to which Kraken2 will write the detailed output of the classification, including the assignment of reads to taxa. output of the classification, including the assignment of reads to taxa
--gzip-compressed	Indicates that the input FASTQ files are compressed with gzip.
--threshold NUM	Value ranges from 0–0.5 can be used to improve precision and sensitivity of genus rank For more info please refer to kraken manual
  b. Visualization using Pavian.
    install.packages(“remotes”)
    remotes::install_github(fbeitwieser/pavian”)
    pavian::runApp(port=5000)
    i. Upload all samples report files into pavian web browser:
      ***Note:*** If the files do not load try to increase upload size using: maxUploadSize = 3000∗1024^2^.

**Figure:**
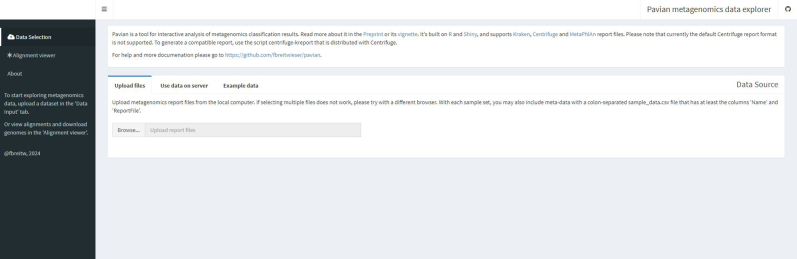
    ii. Results overview.
      The count matrices for all the samples are downloaded in CSV format from the Pavian tool as count_matrix_microbes.csv.

**Figure:**
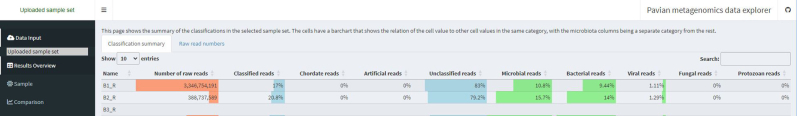
14. Normalization of metagenomic reads (These codes are run in R/Rstudio).
  Normalization is required to account for the differences in sequencing depth across samples, ensuring that variation reflects biological differences rather than technical artefacts. The CSS (Cumulative Sum Scaling) method from the R-package metagenomeSeq is used for this normalization, as it adjusts for varying sample depths by applying median-like quantile normalization.
  a. Creating metagenomeseq object.
    #Set working directory
    getwd()
    setwd(“<path/to/directory>”)
    #Load library
    library(metagenomeSeq)
    #Upload the microbes count matrix
    raw_data<- read.csv(file ="count_matrix_microbes.csv", row.names = 1)
    #create object
    metaSeqObject <- newMRexperiment(raw_data)
    #
    metaSeqObject_CSS <- cumNorm(metaSeqObject, p = cumNormStatFast(metaSeqObject))
    OTU_read_count_CSS <- data.frame(MRcounts(metaSeqObject_CSS, norm=TRUE, log=TRUE))
    View(OTU_read_count_CSS)
    write.csv(OTU_read_count_CSS,”count_matrix_normalized.csv")
    i. The normalized counts csv is next converted into an OTU table, by replacing the taxa name with “OTUn” and saved as OTU_table.

**Figure:**
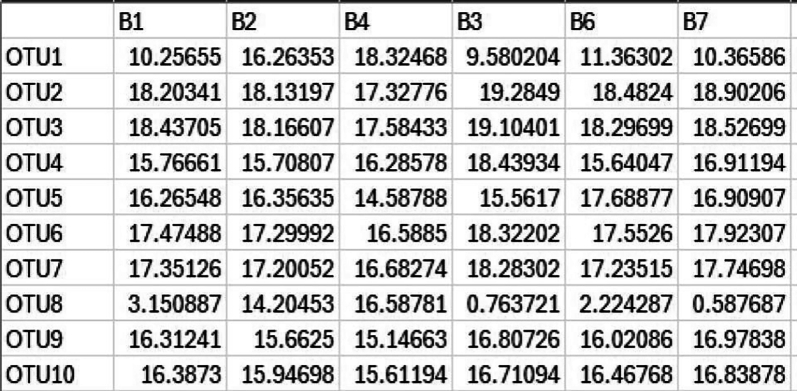
    ii. The taxonomic hierarchies for corresponding OTUs are stored in the taxa file as taxa_table. Using these files, microbial genus with >1% abundance are filtered using the phyloseq R package.

**Figure:**
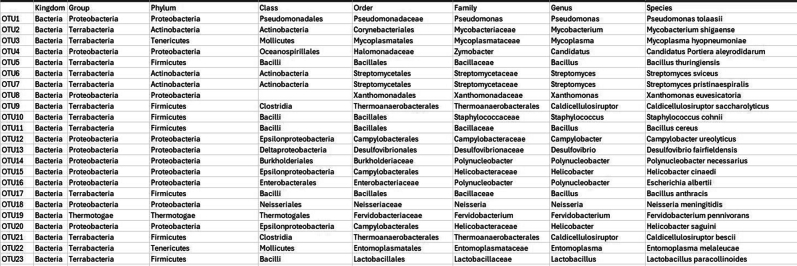
  b. Filtering Taxa with <1% abundance.
    #Load Library
    library("phyloseq")
    library("ggplot2")
    #loading OTU table and taxa files
    otu <- read.csv("OTU_table.csv", sep=",", row.names=1)
    tax <- read.csv("taxa_table.csv", sep=",")
    rownames(tax) <- rownames(otu)
    OTU = otu_table(as.matrix(otu), taxa_are_rows = TRUE)
    TAX = tax_table(as.matrix(tax))
    #import sample information
    sample <- read.csv("sample_table.csv", row.names=1)
    sampledata = sample_data(sample)
    #creating a phyloseq object
    physeq1 = phyloseq(OTU, TAX, sampledata)
    #agglomerating genus of same type
    gp = tax_glom(physeq1, taxrank = "Genus")
    #sorting in descending order
    top <- names(sort(taxa_sums(gp), decreasing = TRUE))
    #computing relative abundance
    gp.prop <- transform_sample_counts(gp, function(x) x / sum(x))
    #removing unwanted OTUs
    gp.prop.top <- prune_taxa(top, gp.prop)
    #combining OTU info, sample info and taxa annotations together
    ps_df <- psmelt(gp.prop.top)
    write.csv(ps_df,"Genus_level_abundance.csv")
    Output: The output file shows the OTU list along with corresponding normalized abundance and Genus.

**Figure:**
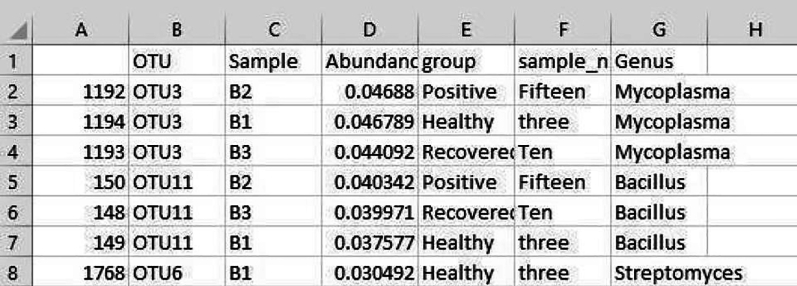
    From the Genus_level_abundance file, we select the microbial genus with >1% abundance and corresponding species taxa from the kraken output file were extracted for further single-cell level analysis.
15. Creating reference index including microbial genomes.
  After selecting the species which we would further like to explore at single-cell level, we will curate a reference genome combining the fasta and gtf of microbial species selected from previous step with human reference fasta and gtf to create an index file for alignment at single-cell level.
  As an example, one microbial species is shown below, similar steps can be done for all the selected microbial species.
  #bacteria genomes
  wget https://ftp.ensemblgenomes.ebi.ac.uk/pub/bacteria/release-59/fasta/bacteria_119_collection/bacillus_cereus_03bb102_gca_000022505/dna/Bacillus_cereus_03bb102_gca_000022505.ASM2250v1_.dna.toplevel.fa.gz</monospace>
  wget https://ftp.ensemblgenomes.ebi.ac.uk/pub/bacteria/release-59/gff3/bacteria_119_collection/bacillus_cereus_03bb102_gca_000022505/Bacillus_cereus_03bb102_gca_000022505.ASM2250v1.59.gff3.gz</monospace>
  a. If the reference genomes are in GFF3 format, first convert them to GTF format.
  b. Place all these files, including the previously used human genome, into a single folder.
  c. Then, concatenate the GTF files into a single genome GTF file and combine the corresponding FASTA files into a single genome FASTA file.
    #Convert gff3 to gtf
    agat_convert_sp_gff2gtf.pl --gff Bacillus_cereus_03bb102_gca_000022505.ASM2250v1.59.gff3 -to Bacillus_cereus.gtf #skip if already in gtf format.
    #creating merged fasta
    cat ∗.fasta > human_microbes.fa.
    #creating merged gtf
    cat ∗.gtf > human_microbes.gtf.
  d. Using the merged gtf and fasta files, a new STAR index file is generated. Ensure all the gtf files are compatible. Check for overlapping annotations using gffcompare or bedtools.
    #Build STAR index.
    STAR --runThreadN 20 --runMode genomeGenerate --genomeDir STAR_index --genomeFastaFiles human_microbes.fa --sjdbGTFfile human_microbes.gtf --genomeSAsparseD 3.
16. Alignment of fastq files.
  Next, we will align the filtered FASTQ files at the single-cell level to generate a count matrix using the new STAR index. You can either use the Seven Bridges platform for BD Rhapsody data or alternatively perform the alignment with STARsolo for both WTA and Abseq data.
  a. Using Seven Bridges platform.
    i. Fastq files of all the samples along with reference genome indexing files in .gz format were uploaded to BD Rhapsody web browser.
      ./sb upload start human_microbes_index.tar.gz --destination <userID>/microbes-run.
      ./sb upload start sample1_R1.fastq.gz --destination <userID>/microbes-run.
      ./sb upload start sample1_R2.fastq.gz --destination <userID>/microbes-run.
    ii. Seven Bridges is an online portal by BD Rhapsody to perform single-cell WTA analysis. Using the default parameters, the updated merged human_microbe STAR index along with raw sequencing reads (fasta files) are uploaded to obtain the cell-specific read count matrix.
      ***Note:*** Complete tutorial on running analysis on Seven Bridges can be found at https://bd-rhapsody-bioinfo-docs.genomics.bd.com/top_introduction.html.
    iii. The output files from Seven Bridges are:
      1. <sample_name>_RSEC_MolsPerCell.csv.
      2. <sample_name>_RSEC_ReadsPerCell.csv.
      3. <sample_name>_DBEC_MolsPerCell.csv.
      4. <sample_name>_DBEC_ReadsPerCell.csv.
      5. <sample_name>_final.BAM.
      6. <sample_name>_final.BAM.bai.
      7. <sample_name>_Expression_Data.st.
      8. <sample_name>_Metrics_Summary.csv.
      9. <sample_name>_UMI_Adjusted_Stats.csv
      ***Note:*** Since Seven Bridges is not an open-source tool, the pipeline used to run on Seven Bridges is also available at this link for performing alignment and count matrix generation manually.
      ***Optional:*** Using STARsolo.
  b. Alternatively, STARsolo can be used to perform alignment as follows:
    #alignment.
    STAR --runThreadN 40 \
     --genomeDir <path/to/STARindex> \
     --readFilesIn read2.fq.gz read1.fq.gz \
     --readFilesCommand zcat \
     --soloType CB_UMI_Complex \
     --soloCBmatchWLtype 1MM \
     --soloCellFilter EmptyDrops_CR \
     --soloCBposition 0_0_0_8 0_13_0_21 0_26_0_34 \
     --soloUMIposition 0_35_0_43 \
     --soloCBwhitelist CL1S.txt CL2S.txt CL3S.txt \
     --outFileNamePrefix <sample_name>/ \
     --outSAMattributes CB UB \
     --outSAMtype BAM SortedByCoordinate
  c. Similarly, run STARsolo for ABseq data which will be used for cell type annotation step later. For Abseq data, an Abseq reference file is required in fasta format. This file can be generated from BD AbSeq Panel Generator (abseq-ref-gen.genomics.bd.com).
    >CD103|ITGAE|AHS0001|pAbO
    AAATAGTATCGAGCGTAGTTAAGTTGCGTAGCCGTT
    >CD161:DX12|KLRB1|AHS0002|pAbO
    GTTATGGTTGTCGGTAGAGTATCGTGTTGCGTTAGT
    ***Note:*** BD Biosciences uses this format for its sequence header: <AntibodyName>|<GeneSymbol>|<SeqID>|pAbO.
  d. Create a gtf file for the fasta file using the following command and create a new STAR index for Abseq data and perform alignment like above.(These codes are run in R/Rstudio).
    # Load required library
    library(GenomicRanges)
    # Define a function to convert FASTA to GTF
    fasta_to_gtf <- function(fasta_file, gtf_file) {
     # Read the FASTA file.
     fasta <- Biostrings::readDNAStringSet(fasta_file, format = "fasta")
    # Extract headers and sequences.
     headers <- names(fasta)
     sequences <- as.character(fasta)
    # Prepare data for GTF file.
     gtf_data <- data.frame(
     seqname = "pAbo", # Assuming single contig; adjust if multiple contigs are present.
     source = "manual",
     feature = "exon", # Feature type; adjust if needed.
     start = rep(1, length(headers)),
     end = nchar(sequences),
     score = ".",
     strand = ".",
     frame = ".",
     attribute = paste0("gene_id \"", headers, "\"; transcript_id \"", headers, "\";"))
    # Write GTF file
    write.table(gtf_data, file = gtf_file, sep = "\t", quote = FALSE, row.names = FALSE, col.names = FALSE)
    }
    # Example usage
    fasta_to_gtf("path/to/input.fasta", "path/to/output.gtf")
  e. The output of STARsolo in Gene/raw will give the:
    i. barcode.csv: contains all the cell indexes one per row.
    ii. features.csv: contains a list of genes detected, one per row with three columns. The first two is the same between WTA and ABseq: <Gene column> <Gene column> <(mRNA) Gene Expression/(Abseq) Antibody Capture).
    iii. matrix.mtx: contains three columns, per row representing first column represents row numbers from features.tsv, second column contains row numbers from barcode.tsv and third column contains the gene counts.
  f. Cell-by-Feature Data Tables.
    #Load library
    library(Matrix)
    getwd()
    setwd("<path/to/directory>")
    # Load STARsolo output files
    mat<− readMM("path_to/matrix.mtx")
    features<− read.delim("path_to/features.tsv", header = FALSE)
    barcodes <- read.delim("path_to/barcodes.tsv", header = FALSE)
    rownames(mat) <- barcodes$V1
    colnames(mat) <- features$V2
    write.csv(as.data.frame(as.matrix(small_matrix)), "<samplename>_RSEC_MolsPerCell.csv")
    #oR
    saveRDS(mat, file = "<samplename>_RSEC_MolsPerCell.rds")
    The output file will look like following: The colnames will contain the gene names and the rownames contain the cell index.

**Figure:**
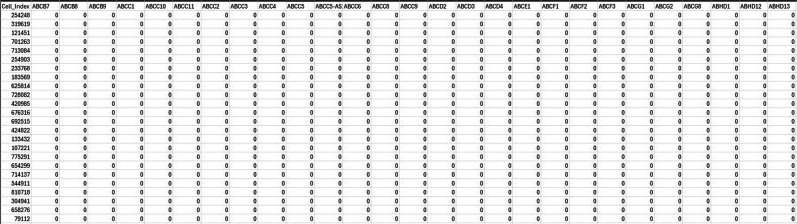
17. Extract microbial read count matrix from the human read count matrix.
  a. Extracting microbial gene count from WTA data.
    From the count matrix downloaded/generated from BD Rhapsody pipeline, we need to extract the cell-specific microbial read counts. For that we need to first curate a list of microbial genes from the gtf file for all the microbes used in the study.
    Here we show it for one microbe, similar steps are performed recursively for all the microbes selected in the study.
    # Load Libraries
    library(data.table)
    library(rtracklayer)
    setwd("path/to/directory")
    #extract all genes list from the genome gtf file
    gtf_files <- list.files( pattern = "∗.gtf")
    for (gtf_file in gtf_files){
    # Import GTF file and convert to a data frame
    gtf.gr = rtracklayer::import(gtf_file)
    gtf.df = as.data.frame(gtf.gr)
    # Extract unique gene IDs
    genes = unique(gtf.df[ , "gene_id"])
    genes <-as.data.frame(genes)
    # Create output filename based on the GTF file
    output_file <- paste0(sub(".gtf$", "", basename(gtf_file)), "_genes.csv")
    write.csv(genes, file = output_file, row.names = FALSE)
    print(paste("Processed:", gtf_file))
    }
    ***Note:*** Make sure the gtf file is complete without any missing values.

**Figure:**
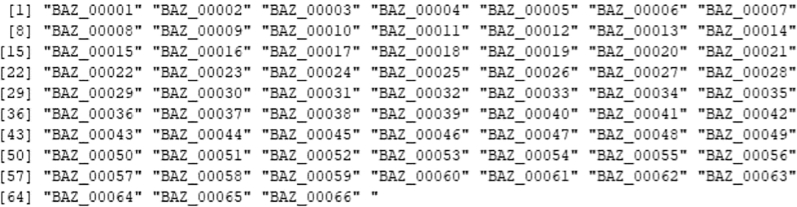
  b. Next, we will segregate the human, microbial, and antibody genes vis-a-vis cells count matrix from the combined read count matrix obtained from BD Rhapsody pipeline run previously. To do so, we will first annotate all the microbial reads with specific species names.
    Here, we are taking Bacillus sp as an example to demonstrate. It can be followed for all the combined microbe gene names similarly.
    #Load Libraries
    library(data.table)
    library(dplyr)
    library(tidyverse)
    #upload the read count matrix of human, antibody and microbes that is generated by BD Rhapsody platform for each sample.
    cc_sample1 <- fread("B1_RSEC_MolsPerCell.csv")
    #OR.
    #Alternatively load RDS file saved from STARsolo alignment
    cc_sample1 <-loadRDS("<samplename>_RSEC_MolsPerCell.rds")
    #Get gene list from the count matrix:
    countmat_gene_list1 <- colnames(cc_sample1)
    >countmat_gene_list1.

**Figure:**
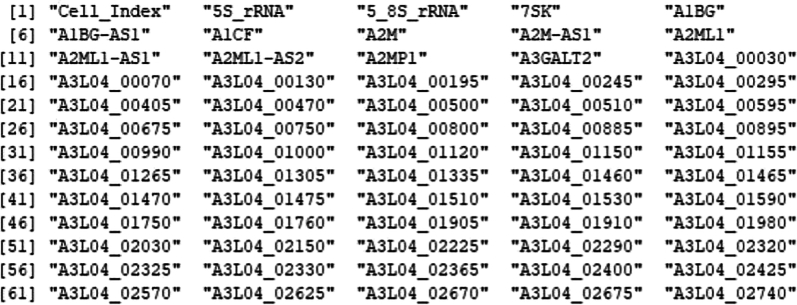
  c. We have loaded the count matrix for one sample named B1_RSEC_MolsPerCell. We will now load the microbial gene list curated from gtf in the previous step.
    #renaming the column name
    cc_sample1 <- t(cc_sample1)
    colnames(cc_sample1) <- cc_sample1[1,]
    cc_sample1 <- cc_sample1[-1,]
    #upload microbial gene list
    microbe_gl <- as.vector(read.csv("path/to/BAnthracis_genes.csv"))
    #check if these genes are there in the raw data
    genes_microbe_gl_found <- intersect(countmat_gene_list1, microbe_gl$genes)
    #number of microbial genes found in count matrix
    length(genes_microbe_gl_found)
  d. Now, we will subset the rows with overlapping microbial genes.
    #subset original/raw data based on above pathogen genes
    microbe_gl_mat <- cc_sample1[row.names(cc_sample1) %in% genes_microbe_gl_found, ]
    # Get read count for that particular pathogen species
    microbe_gl_mat <- as.data.frame(microbe_gl_mat)
    dim(microbe_gl_mat)
    col<-ncol(microbe_gl_mat)
  e. Merge individual gene list to per microbial gene count.
    #concatenate all the genes in a single column
    microbe_gl_mat_sum <- colSums(microbe_gl_mat[, 1:col])
    BAnthracis_gl_mat_sum <-as.data.frame(microbe_gl_mat_sum)
    BAnthracis_gl_mat_sum <- t(BAnthracis_gl_mat_sum)
    dim(BAnthracis_gl_mat_sum)
    BAnthracis_gl_mat_sum <-as.data.frame(BAnthracis_gl_mat_sum)
    #Save the row name as that microbial species name
    rownames(BAnthracis_gl_mat_sum) <- "Baccillus_Anthracis"
  f. Combine all the sample-specific per cell count matrix for microbes from different samples as a single combined count matrix (microbial_counts_samplewise.csv) for performing differential microbial abundance analysis.
    #concatenate all the microbial read count matrix
    sample1_mat<-list(BAnthracis_gl_mat_sum,Bthurigenesis_mat_sum, Bdutonii_mat_sum, Tgeofonti_mat_sum)
    #combined all the microbial row
    sample1_mat <- do.call(rbind, sample1_mat)
    # Write the combined matrix to a CSV file
    write.csv(sample1_mat , "sample1_microbe_count_matrix.csv")
18. Extracting counts from Abseq count matrix.
  a. Next, we will separate the counts for Abseq to perform cell annotations. For this, we will require the list of all the antibodies. We will follow a similar method as above to subset and annotate Abseq data and save the count matrix.
    #Extract antibody read count matrix
    all_antibody_genes <- as.vector(read.csv("antibody_list.txt"))
    # check if these genes are there in the gene list
    all_antibody_genes_found <- intersect(countmat_gene_list1, all_antibody_genes$Genes)
    # number of genes
    length(all_antibody_genes_found)
    #subset original/raw data based on above pathogen genes
    abseq_mat <- cc_sample1[row.names(cc_sample1) %in% all_antibody_genes_found, ]
    #Total Read Count Value of the matrix
    sum(abseq_mat)
    # save antibody count matrix in CSV file
    write.csv(abseq_mat,"sample1_abseq_count_matrix.csv")
  b. Since the Abseq and microbial rows are annotated, the remaining counts belong to the human transcriptome, thus we will extract genes excluding these for cell annotation.
    #Extract count matrix excluded microbes and antibody genes.
    #Read all combined pathogenic genes
    all_microbes_genes <-as.vector(read.csv("all_microbes_genes.txt"))
    all_microbes_genes_found <- intersect(countmat_gene_list1,all_microbes_genes$Genes)
    # Number of Non-zero values in matrix
    length(all_microbes_genes_found)
    #exclude microbial genes
    microbes_only_sample1 <- cc_sample1[!row.names(cc_sample1) %in% all_microbes_genes_found, ]
    dim(microbes_only_sample1)
    colnames(microbes_only_sample1) <- microbes_only_sample1[1,]
    microbes_only_sample1 <- microbes_only_sample1[-1,]
    dim(microbes_only_sample1)
    #exclude antibody genes.
    Human_wta <- microbes_only_sample1[!row.names(microbes_only_sample1) %in% all_antibody_genes_found, ]
    dim(human_wta)
    #save the human count matrix
    write.csv(human_wta,"sample1_human_count_matrix.csv")
    #Do the same for all samples.
19. Cell Clustering and Annotation.
  To identify the cell type, we will use the Abseq and WTA files from previous steps to cluster and classify cell-types based on gene abundance and surface marker expression value for each sample.
  a. Cell Clustering and Annotation.
    # Load libraries
    library(Seurat)
    library(dplyr)
    library(sctransform)
    library(data.table)
    #First, read the count matrix for human transcriptome, repeat for all samples
    sample1_human_wta <- read.csv("path/to/dir/sample1_human_count_matrix.csv", row.names = 1)
    The loaded file contains the cell index as the column name and gene names as row names.

**Figure:**
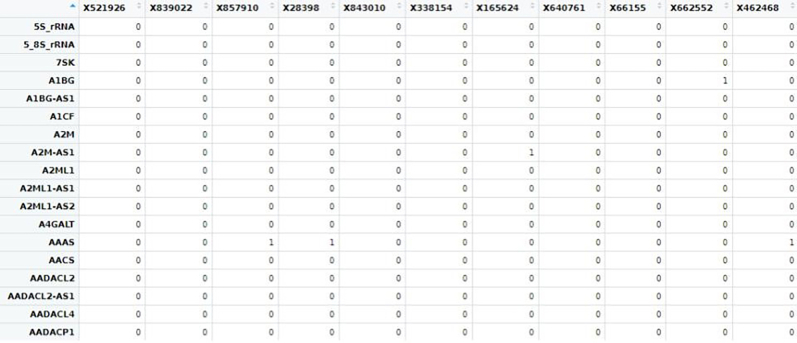

    #Next, read the count matrix for the AbSeq; repeat for all samples.
    sample1_abseq_mat <- read.csv("path/to/dir/sample1_abseq_count_matrix.csv", row.names = 1)

**Figure:**
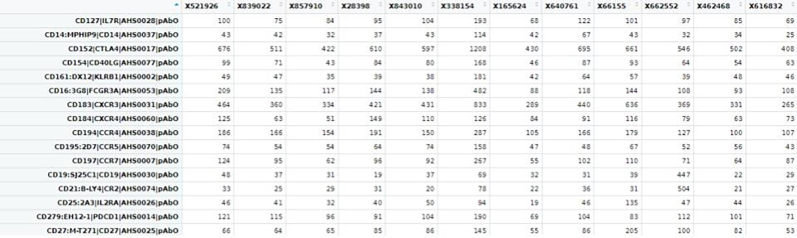

    #Finally, read the count matrix for the pathogens, repeat for all samples
    sample1_microbe_mat <- read.csv("/path/to/dir/sample1_microbe_count_matrix.csv", row.names = 1)
    The row names contain the gene/species names and columns contain cell index. The cell index will be the same in all three count matrices.

**Figure:**
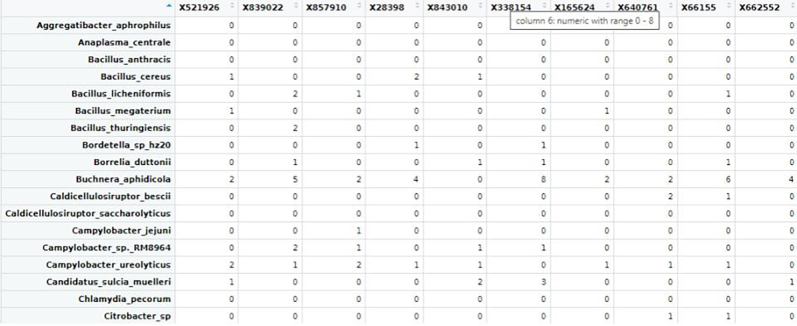

    #Create a SeuratObject using the human WTA counts, and add the AbSeq and pathogen counts as AssayObject. Repeat for all samples
    sample1 <- CreateSeuratObject(counts = sample1_human_wta)
    sample1_abseq <- CreateAssayObject(count = sample1_abseq_mat)
    sample1_microbe <- CreateAssayObject(count = sample1_microbe_mat)
    sample1 [["abseq"]] <- sample1_abseq
    sample1 [["microbe"]] <- sample1_microbe.

**Figure:**
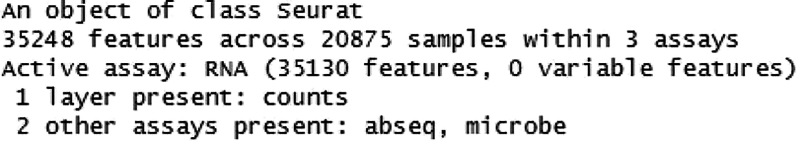
  b. Once a Seurat object is created, cells should be filtered based on key quality metrics such as the number of features (genes) detected per cell and the percentage of mitochondrial reads. The filtering criteria can be optimized for the specific dataset, but typically, cells with more than 20% mitochondrial reads and with a low number of captured genes are excluded from further analysis (Figure 1).
    #Add labels defining the sample and group to each SeuratObject
    sample1$sample <- "S1"
    sample1$group <- "group1"
    #Remove low quality cells, cells containing very low and high numbers of unique transcripts. Repeat for all samples
    sample1 [["percent.mt"]] <- PercentageFeatureSet(sample1, pattern = "ˆMT.")
    #the cut-off parameters should be optimized based on the dataset
    sample1<− subset(sample1, subset = nFeature_RNA > 200 & nFeature_RNA < 3000 & percent.mt < 20)

**Figure 1:**
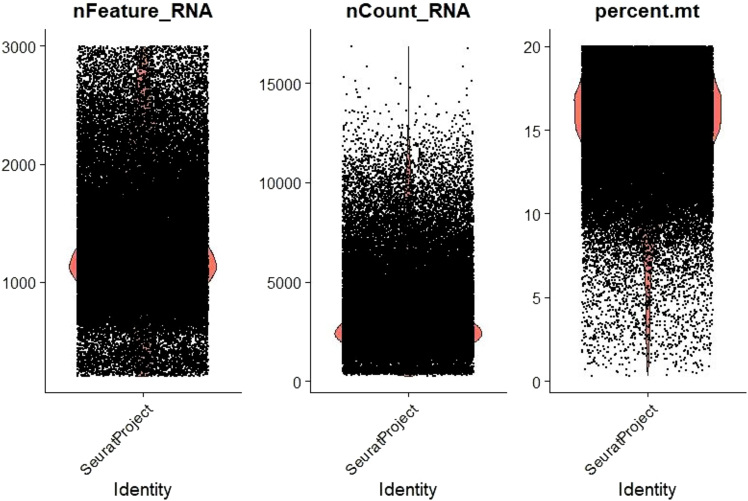
Quantitative summary of scRNA-seq data Barplots with jitter showcasing the number of unique RNA reads captured per cell (nfeature_RNA), total RNA counts (nCount_RNA), and the percentage of mitochondrial RNA (percent.mt) across samples.
    The number of unique genes (nFeature_RNA), number of total genes (nCount_RNA) and percentage of mitochondrial genes (percent.mt) are plotted to visualize their distribution after applying the above filtering criteria.
  c. Once Seurat objects are created for each individual sample, they can be merged together and saved as an RDS file which can be used for further downstream clustering and annotation.
    #Merge all the sample level SeuratObject into one single object
    covid <- merge(sample1, c(sample2,sample3,...), add.cell.ids = c("sample1″, " sample2″, " sample3″,...))
    Seurat::Project(object = covid) <- 'covid'
  d. The merged object now contains the QC-passed cells from each samples. The gene expression data is stored in the RNA assay, while the surface marker expression and microbial abundance are stored in the abseq and microbe assay respectively.

**Figure:**
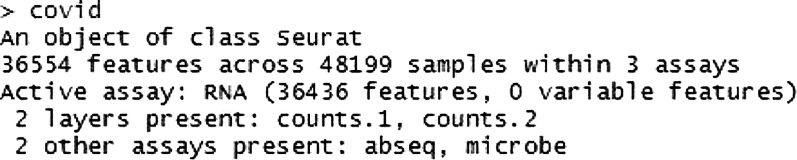
20. Visualization of Annotated clusters.
  a. We next perform integration, normalization, and dimensionality reduction of the data, followed by clustering analysis. First, SCTransform is applied to normalize and correct for batch effects within individual samples, ensuring that differences in sequencing depth and technical variations are addressed. Next, SelectIntegrationFeatures and PrepSCTIntegration are used to prepare features for integration across multiple datasets, followed by FindIntegrationAnchors and IntegrateData to harmonize the datasets for downstream analysis.
    #Apply data normalization and batch effect correction
    covid <- SplitObject(covid, split.by = " sample1″)
    covid <- lapply(X = covid, FUN = SCTransform)
    #Perform the data integration
    features <- SelectIntegrationFeatures(object.list = covid, nfeatures = 5000)
    covid <- PrepSCTIntegration(object.list = covid, anchor.features = features)
    covid.anchors <- FindIntegrationAnchors(object.list = covid, normalization.method = "SCT", anchor.features = features)
    covid.sct <- IntegrateData(anchorset = covid.anchors, normalization.method = "SCT")
    #Dimension Reduction
    covid.sct <- RunPCA(covid.sct, verbose = TRUE)
    DimPlot(covid, reduction = "pca", raster = FALSE, group.by = "orig.ident") + NoLegend()
    ElbowPlot(covid.sct) #Determine the dimensionality of the data
    covid.int.sct <- RunTSNE(covid.int.sct, dims = 1:20, reduction = "pca")
    For dimensionality reduction, PCA is applied to reduce the complexity of the data, followed by ElbowPlot to determine the optimal number of principal components (Figure 2).

**Figure 2:**
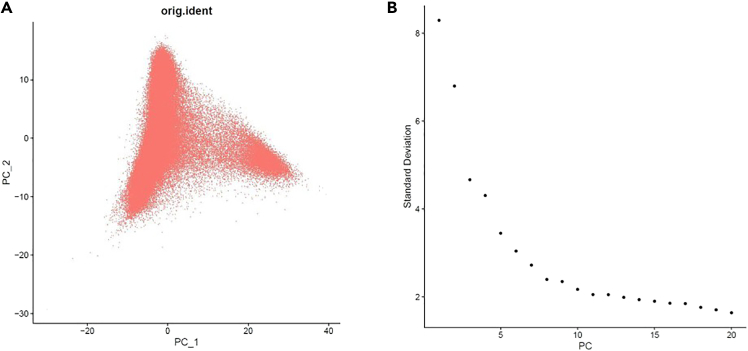
Principal component analysis and optimal dimensionality selection (A) Principal Component Analysis (PCA) plot. (B) Elbow plots identifying the optimal number of principal components for dimensionality reduction.
    The precomputed principal components as optimized through the PCA and Elbow plot were used for t-SNE visualization of the integrated data in a lower-dimensional space (Figure 3). Lastly, FindNeighbors and FindClusters identify cell populations by clustering cells based on their gene expression profiles, optimizing the clustering resolution as per the data’s characteristics (Figure 4).

**Figure 3:**
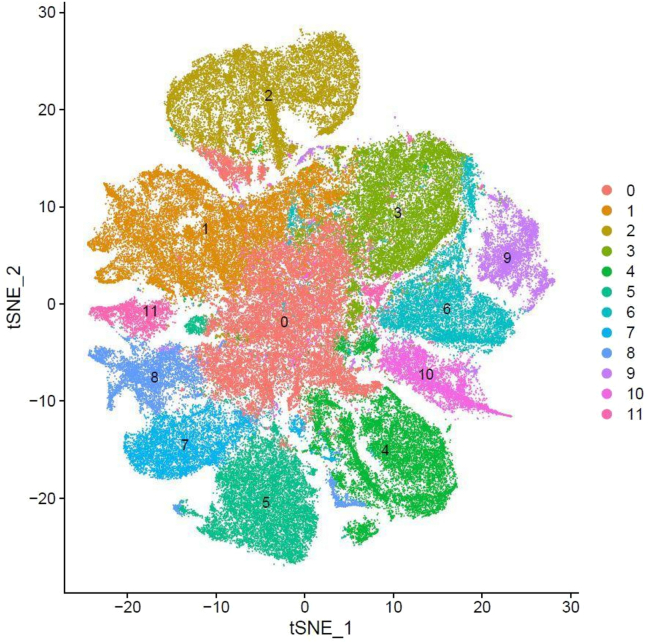
Visualization of cellular clusters by t-SNE.t-SNE plot illustrating distinct cell clusters based on transcriptomic profiles

**Figure 4:**
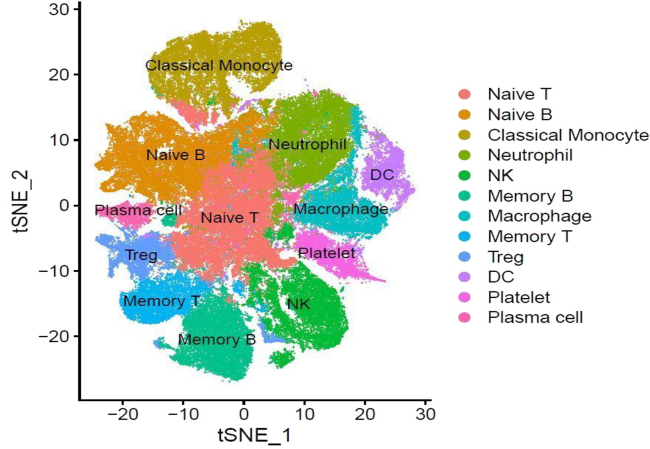
Annotation of cell clusters visualized by t-SNE t-SNE plot highlighting annotated cell clusters.
  b. This workflow enables the analysis of heterogeneous cell populations in a multi-sample single-cell dataset. (See Table 14.)
    #Identifying nearest neighbors and clustering of data
    covid.int.sct <- FindNeighbors(covid.int.sct, reduction = "pca", dims = 1:20)
    covid.int.sct <- FindClusters(covid.int.sct, resolution = 0.4)
    #optimize the resolution according to the data.
    #Visualization of the clusters.
    DimPlot(covid.int.sct, reduction = "tsne", repel = TRUE, raster = FALSE)
    DimPlot(covid.int.sct, reduction = "tsne", split.by = "sample", raster = FALSE)
    DimPlot(covid.int.sct, reduction = "tsne", split.by = "group", raster = FALSE)
    #Identify cluster-specific gene expression pattern
    covidintmarkers <- FindAllMarkers(covid.int.sct, only.pos = TRUE, min.pct = 0.25, logfc.threshold = 0.25) #min.pct and logfc.threshold to be optimized as per data

**Figure:**
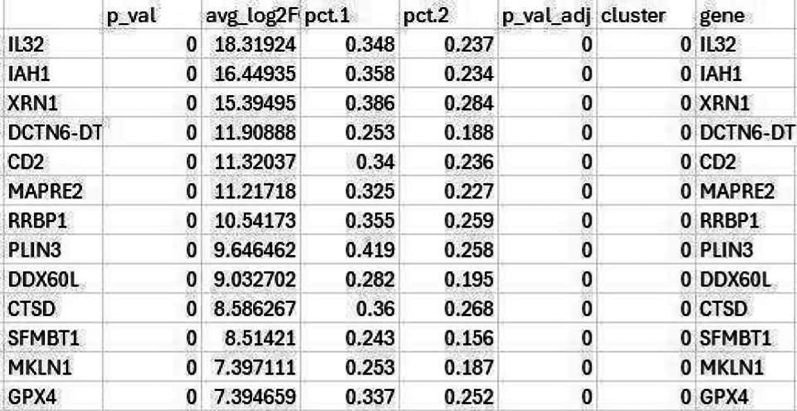

    #Manually annotate the clusters based on cluster-specific gene expression pattern, and then add the cell type labels to the cluster
    new.cluster.ids <- c("Naive T", "Naive B", "Classical Monocyte", "Neutrophil", "NK", "Memory B", "Macrophage", "Memory T", "Treg", "DC", "Platelet", "Plasma cell")
    names(new.cluster.ids) <- levels(covid.int.sct)
    covid.int.sct <- RenameIdents(covid.int.sct, new.cluster.ids)
    DimPlot(covid.int.sct, reduction = "tsne", label = TRUE, pt.size = 0.5, raster = FALSE) + NoLegend()
    #save the annotated count matrix as an RDS file
    saveRDS(covid)

**Table 14:** Gene list used for manual annotation of the clusters

Cluster	Cell type	Marker genes
Cluster 0	Naïve T	CD45RA, CCR7, CD3E, CD62L, IL7R
Cluster 1	Naïve B	CD19, CD21, CD24, CD40
Cluster 2	Classical Monocyte	CD14, CD16, CD64, A100A4, LYZ
Cluster 3	Neutrophil	CD15, CD16, CD32, CD33
Cluster 4	NK	GNLY, NKG7
Cluster 5	Memory B	CD19, MS4A1, CD21, CD27
Cluster 6	Macrophage	ADGRE1, CD68, CD163, CCR5, TLR2, TLR4
Cluster 7	Memory T	IL7R, S100A4, CD45RO, IL2RA, CD62L, CD8A, CD4, CD3E
Cluster 8	Treg	CD3E, CD4, CD25, CD127, CTLA4
Cluster 9	DC	FCER1A, CST3
Cluster 10	Platelet	PPBP
Cluster 11	Plasma Cell	CD27, CD38, CXCR4
21. Microbial Cell filtration.
  The annotated cell types can now be utilized to assess the abundance of different microbial species in a cell-type-specific manner and to conduct metagenomic analysis.
  a. Extract the microbial gene counts from the microbe assay.
  b. Define a threshold for high microbial abundance (e.g., top 10%).
  c. Identify the cells with high microbial counts (in this case we > 5 was considered but it depends on the data).
  d. Calculate how many of these cells belong to each cell type and what percentage of each cell type they represent.
    This analysis provides insight into which cell types show significant microbial gene expression.
    # Load library
    library(Seurat)
    # **Normalize the data in the "pathogen" assay**
    covid2 <- NormalizeData(covid, assay = "pathogen")
    # Subset the Seurat object to include cells with more than 5 features in the "pathogen" assay
    subset <- subset(covid2, subset = nFeature_pathogen > 5)
    # Print the number of cells before subsetting
    cat("Number of cells before subset:", ncol(covid2), "\n")
    # Print the number of cells after subsetting
    cat("Number of cells after subset:", ncol(subset), "\n")
    # **Print the number of cells removed during subsetting**
    cat("Number of cells removed:", ncol(covid2) - ncol(subset), "\n")
    Number of cells before subset: 114704.
    Number of cells after subset: 30687.
    Number of cells removed: 84017.
  e. When you run SCTransform() on a Seurat object, it stores multiple models internally to handle differences in sequencing depth across cells or conditions. These models account for variable library sizes, batch effects, and other factors. The PrepSCTFindMarkers() function aligns the different models and prepares the data in a format that can be used by FindMarkers().
    # Display the levels (cell identities)
    levels(subset)
    #
    subset<− PrepSCTFindMarkers(subset)
    # Find markers (differentially expressed genes) in the subsetted dataset.
    FindMarkers(subset, ident.1 = "Neutrophil_infected", ident.2 = "Neutrophil_recovered", logfc.threshold = 0, test.use = "wilcox", min.pct = 0.1)
  f. The FindMarkers() function in Seurat is used to identify differentially expressed genes between two groups of cells. Typically, it compares two cell identities (e.g., cell types, conditions, or clusters) and returns genes that are significantly different in expression between the two groups.
  g. The output of FindMarkers() is a data frame, where each row represents a gene. The columns include important information such as avg_log2FC, which provides the average log2 fold-change in expression between the two groups, and p_val, the p-value from the differential expression test.
  h. The results are also adjusted for multiple testing, with the adjusted p-value presented as p_val_adj (using FDR correction). Moreover, pct.1 and pct.2 indicate the percentage of cells expressing the gene in “Neutrophil_Healthy” and “Neutrophil_Positive,” respectively.

**Figure:**
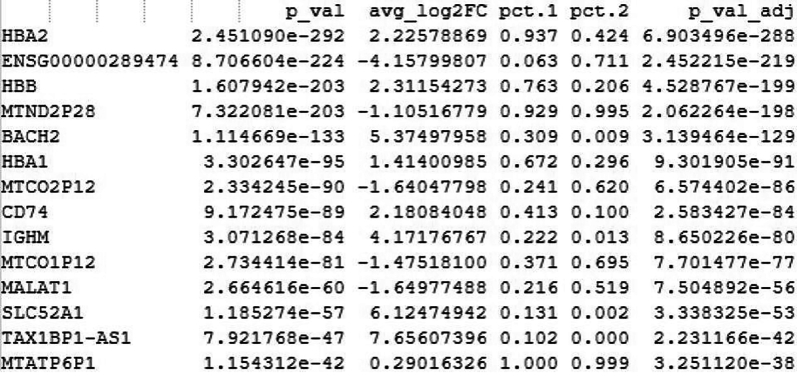
  i. Cell counts with microbial reads captured.
    # Identify unique cell types in the subsetted Seurat object
    cell_types <- unique(Idents(subset))
    # Count the number of cells in each cell type
    cell_type_counts <- table(Idents(subset))
    # Print the cell type-wise count of cells
    print(cell_type_counts)
    > cell_type_counts

**Figure:**
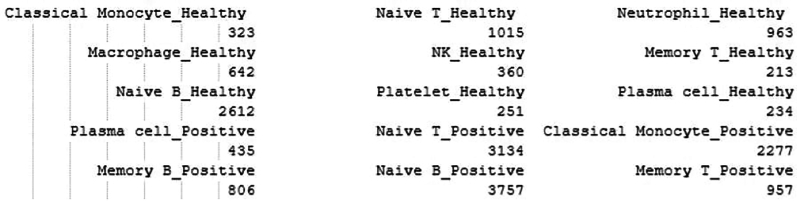
22. Differential Microbial Abundance Analysis at pseudo-bulk level.
  Using the cell-specific microbial counts for each sample, we will perform differential abundance of microbes in different cell-types. We will first process the single-cell data by aggregating the counts by cell-type and sample, then prepare corresponding metadata, and perform differential expression analysis using DESeq2. Using the counts and metadata saved in the COVID RDS file in previous steps, we will perform the following steps.
  a. Aggregating counts by cell type.
    # Load libraries
    library(tidyverse)
    library(cowplot)
    library(edgeR)
    library(Matrix)
    library(reshape2)
    library(S4Vectors)
    library(SingleCellExperiment)
    library(pheatmap)
    library(apeglm)
    library(png)
    library(DESeq2)
    library(RColorBrewer)
    library(data.table)
    library(Matrix.utils)
    #loading count data
    counts<-read.csv("microbial_counts_samplewise.csv", row.names = "X")
    counts<-counts[,c(-1,-2)]
    t_counts<-t(counts)
    #loading metadata
    metadata<-read.csv("microbial_counts_metadata.csv", row.names = "X")
    metadata[,'celltype']<-as.factor(metadata[,'celltype'])
    metadata[,'sample']<-as.factor(metadata[,'sample'])
    metadata[,'group']<-as.factor(metadata[,'group'])
    str(metadata)
    The metadata file consists of 3 factors: Celltype, Sample and Group.

**Figure:**
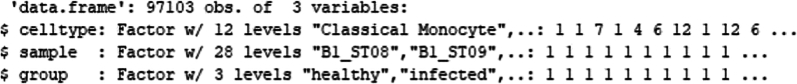

    #Create SingleCellExperiment object
    sce<-SingleCellExperiment(assays = list(counts = counts), colData = metadata)
    #Extract unique cluster and sample names
    cluster_names <- levels(colData(sce)$celltype)
    sample_names <- levels(colData(sce)$sample)
    #Subset metadata for aggregation
    groups <- colData(sce)[, c("celltype", "sample")]
    head(groups)
    >sce

**Figure:**
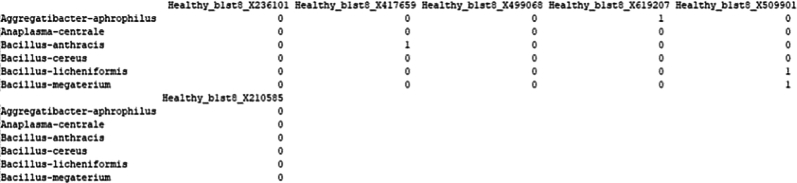
    >group

**Figure:**
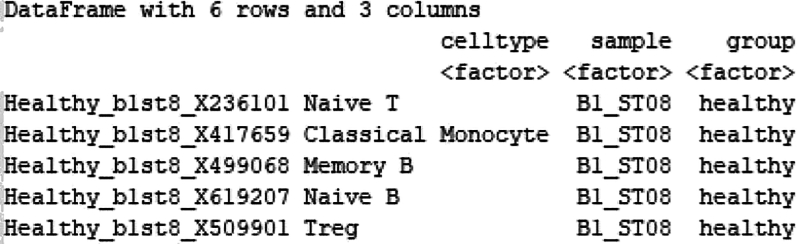

    #Aggregating counts across cluster-sample groups.
    #transposing row/columns to have cell_ids as row names matching those of groups
    aggr_counts <- aggregate.Matrix(t(counts(sce)), groupings = groups, fun = "sum")
    aggr_counts <- t(aggr_counts)
    #Exploring structure of function output
    tstrsplit(colnames(aggr_counts), "_") %>% str()
    #Comparing the first 10 elements of our input and output strings
    head(colnames(aggr_counts), *n* = 10)
    head(tstrsplit(colnames(aggr_counts), "_")[[1]], *n* = 10)
    #Extract indices of columns matching a specific cell type
    b_cell_idx <- which(tstrsplit(colnames(aggr_counts), "_")[[1]] = = "Naive B″)
    b_cell_idx
    #Loop over all cell types to extract corresponding counts, and store information in a list.
  b. Preparing Metadata for DE Analysis
    i. We need to organize metadata and count data for differential expression (DE) analysis by clusters of cells. It splits the aggregated counts matrix based on cell clusters, retrieves the corresponding metadata, and tracks the number of cells per sample and cluster. The result is a list (metadata_ls), where each element contains the metadata and cell count information for a specific cluster, making it ready for DE analysis or further downstream processing.
    ii. We will create a list, counts_ls, where each element contains a subset of the aggregated counts matrix (aggr_counts) based on the corresponding cluster (cell type) name from cluster_names. The tstrsplit function splits the column names of aggr_counts to extract cluster names (the first element in the split) and then indexes the matrix.
      #Initialise list for counts by cluster
      counts_ls <- list()
      for (i in seq_along(cluster_names)) {
      column_idx <- which(tstrsplit(colnames(aggr_counts), "_")[[1]] = = cluster_names[i])
      counts_ls[[i]] <- aggr_counts[, column_idx]
      names(counts_ls)[i] <- cluster_names[i]
      }
    iii. Next, we will prepare the metadata dataframe from the SingleCellExperiment object (sce). It selects the relevant columns (group and sample) for later differential expression (DE) analysis and removes any duplicate rows based on sample. The sample column is then used as row names.
      #Explore the different components of the list
      str(counts_ls)
      #Prepare metadata for DE analysis
      metadata <- colData(sce) %>%
      as.data.frame() %>%
      dplyr::select(group, sample)
      #Exclude duplicate rows
      metadata <- metadata[!duplicated(metadata), ]
      rownames(metadata) <- metadata$sample
      head(metadata)
    iv. This loop initializes the metadata_ls list for storing metadata for each cluster. It begins by creating a data frame (df) where the column names of the cluster-specific counts (counts_ls) are split into cell type, batch, and sample IDs. These identifiers are used to track and merge sample information.
      # Number of cells per sample and cluster
      t <- table(colData(sce)$sample, colData(sce)$celltype)
      #Initialise list for metadata by cluster
      metadata_ls <- list()
      for (i in 1:length(counts_ls)) {
      #Initiate a data frame for cluster i with one row per sample (matching column names in the counts matrix)
      df <- data.frame(cluster_sample_id = colnames(counts_ls[[i]]))
      #Use tstrsplit() to separate cluster (cell type) and sample IDs
      df$celltype <- tstrsplit(df$cluster_sample_id, "_")[[1]]
      df$batch <- tstrsplit(df$cluster_sample_id, "_")[[2]]
      df$sample <- tstrsplit(df$cluster_sample_id, "_")[[3]]
      df$sample_id<-paste0(df$batch,"_",df$sample)
      df$group<-metadata$group[match((df$sample_id), row.names(metadata))]
    v. Now we need to retrieve cell count information from the global count table t, by matching it to the correct sample IDs, and append it to the df dataframe. Only samples with non-zero cell counts for the current cluster are retained.
      #Retrieve cell count information for this cluster from global cell count table
      idx <- which(colnames(t) = = unique(df$celltype))
      cell_counts <- t[, idx]
      #Remove samples with zero cell contributing to the cluster
      cell_counts <- cell_counts[cell_counts > 0]
      #Match order of cell_counts and sample_ids
      sample_order <- match(df$sample_id, names(cell_counts))
      cell_counts <- cell_counts[sample_order]
      #Append cell_counts to data frame
      df$cell_count <- cell_counts
    vi. The cluster-specific metadata (df) is joined with the generic metadata table to form a complete metadata dataframe for the current cluster. The plyr::join() function ensures that the metadata from both dataframes is merged based on shared column names. Finally, the resulting metadata for the cluster is stored in metadata_ls.
      #Join data frame (capturing metadata specific to cluster) to generic metadata
      df <- plyr::join(df, metadata,
      by = intersect(names(df), names(metadata)))
      #Update rownames of metadata to match colnames of count matrix, as needed later for DE
      rownames(df) <- df$cluster_sample_id
      #Store complete metadata for cluster i in list
      metadata_ls[[i]] <- df
      names(metadata_ls)[i] <- unique(df$celltype)
      }
      #Explore the different components of the list
      str(metadata_ls)

**Figure:**
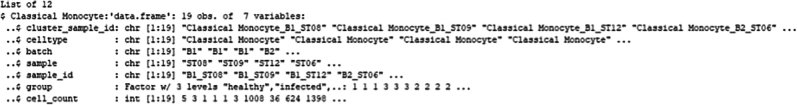
  c. Pseudo Bulk Analysis Using DESeq2.
    The pseudobulk approach used here aggregates single-cell data into bulk samples based on cell type, allowing for robust statistical analysis of differential expression across conditions. Visualization steps such as PCA and heatmaps help in understanding the data structure and relationships (Figure 5).
    #Starting pseudo bulk analysis using DESEq2#
    # Select cell type of interest
    cluster_names
    # Double-check that both lists have same names
    all(names(counts_ls) = = names(metadata_ls))
    idx <- which(names(counts_ls) = = "DC")
    cluster_counts <- counts_ls[[idx]]
    cluster_metadata <- metadata_ls[[idx]]

**Figure:**
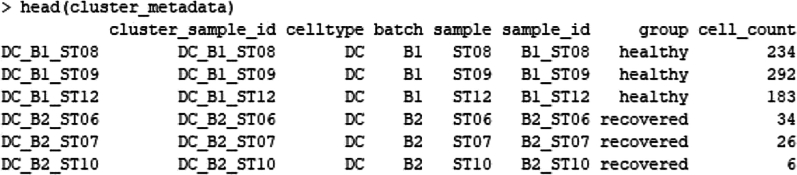

    # Check matching of matrix columns and metadata rows
    all(colnames(cluster_counts) = = rownames(cluster_metadata))
    cluster_counts<-as.matrix(cluster_counts +1)#adding 1 to the count matrix to allow log transformation of the data.
    # Create DESeq2 object
    dds <- DESeqDataSetFromMatrix(cluster_counts,
    colData = cluster_metadata,
    design = ∼ group)
    # Transform counts for data visualization
    rld <- rlog(dds, blind = TRUE)
    # Plot PCA.
    DESeq2::plotPCA(rld, intgroup = "cell_count")
    # Extract the rlog matrix from the object and compute pairwise correlation values
    rld_mat <- assay(rld)
    rld_cor <- cor(rld_mat)
    # Plot heatmap
    pheatmap(rld_cor, annotation = cluster_metadata[, c("group"), drop = F])
    # Run DESeq2 differential expression analysis
    dds <- DESeq(dds)
    res <-results(dds)

**Figure:**
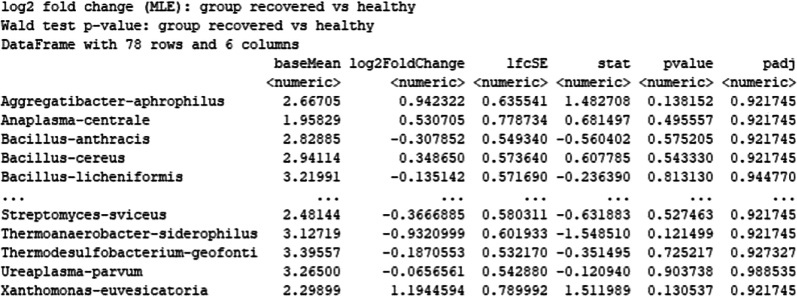

**Figure 5:**
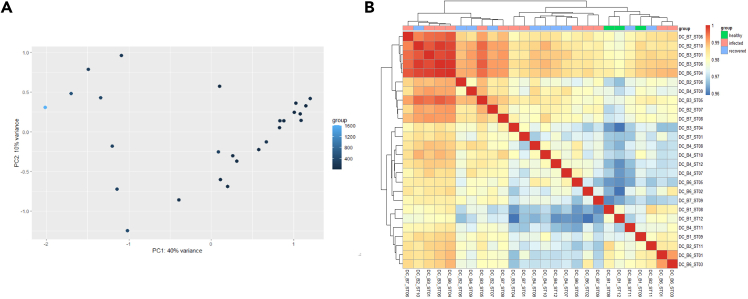
Microbial read distribution and normalization across samples (A) PCA plot showing distribution of cell counts with microbial reads across samples. The scale represents the cell count. (B) The heatmap on the bottom illustrates the correlation of gene expression levels across all pairwise combinations of samples in the dataset. The hierarchical tree represents similarity between the samples, and the colored bars on top represent group information (green, healthy; pink, infected; blue, recovered). The gradient scale represents the correlation score.

## Expected outcomes

The major outcome of this protocol involves the identification of cell-type specific microbial transcripts and their possible role in differential disease severity. Through an integrative multi-omics approach of scRNA-seq and metagenomic analysis, this method provides new insights into intracellular microbial dynamics. It offers an in-depth understanding of microbial genus and species composition, as well as their interactions and functional roles within the host cells. This method offers an effective means of identifying diverse intracellular microbial genus/species, their association with definitive cell types and their effect on disease phenotypes.

## Limitations

The scRNA-seq protocol is based on poly-A capture of transcripts, which is less abundant in microbes. As compared to the host the amount of mRNA captured from microbes is limited. This offers an ideal opportunity to include a diverse range of methods to capture the mRNA lacking poly-A. However, it’s important to consider that the microbial genome, especially that of bacteria consisted of >80% rRNA and 5%–10% mRNA, so the chances of capturing rRNA content are very high. This is even more important as we undertake integrative analysis for the expressed host genes and the transcribed microbes in those cohorts of patients.

## Troubleshooting

### Problem 1

Low sample volume.

### Potential solution

• Extra washing steps can be omitted.
• The temperature mentioned for each specific step should be strictly followed.

### Problem 2

PCR2 product yield is too low.

### Potential solution

• Ensure correct primers and correct thermocycler programs are used.
• Use the specified volume of AMPure XP beads.
• Use only specified volume and concentration of ethanol.

### Problem 3

No or low yield of RPE-PCR.

### Potential solution

• Repeat RPE from beads again.
• Repeat PCR with RPE product for 4–5 more cycles.

### Problem 4

Bioanalyzer peaks do not match.

### Potential solution

• The washing step can be repeated to remove primer dimers or.
• PCR cycles in the prior can be repeated with low or high number of cycles depending upon the bioanalyzer profile.

### Problem 5

If during installation of the package, there is any error.

### Potential solution

• Check the format of the command and try to install packages individually. or upgrade R to the latest version. 4.3 or higher.

## Resource availability

### Lead contact

Further information and requests for resources and reagents should be directed to and will be fulfilled by the lead contact, Rajesh Pandey (rajesh.p@igib.res.in).

### Technical contact

Technical information inquiries should be directed to and will be fulfilled by the technical contact Rajesh Pandey (rajesh.p@igib.res.in).

### Materials availability

This study did not generate new unique reagents and materials.

### Data and code availability

• The accession number for the scRNA-seq data reported in this paper is submitted to the NCBI GEO database: GSE201088. The codes used in this protocol are also available at GitHub: https://github.com/INGEN-HOPE/Single-cell-microbial-Analysis.
• This paper does not report the original code.
• Any additional information required to reanalyze the data reported in this paper is available from the lead contact upon request.

## Acknowledgments

The authors duly acknowledge all the COVID-19 patients and healthy and recovered individuals who participated in the study. The authors also would like to acknowledge the support of Dr. D. Y. Patil Medical College, Hospital & Research Centre, Kolhapur, Maharashtra, India, for providing the relevant samples for this study. The authors acknowledge the help and support from Dr. Bharti Kumari and Dr. Aradhita Baral toward facilitation as research manager and coordination with the funders. The authors acknowledge the support of Anil Kumar and Nisha Rawat toward COVID-19 sample transport and sample management. J.S. acknowledges UGC for her research fellowship. P.C. acknowledges the CSIR for their research fellowship. This research was funded by the Bill and Melinda Gates Foundation, grant numbers INV-033578 and INV-030592.

## Author contributions

P.C. performed the experiments; S.Y., P.M., and P.C. performed analysis; P.C., P.M., J.S., and R.P. wrote the manuscript; R.P. arranged funding and coordinated partnership with the hospital. All authors contributed to the article and approved the submitted version.

## Declaration of interests

The authors declare no competing interests.
